# Non-invasive imaging of defence responses in plants

**DOI:** 10.1038/s41467-026-70075-1

**Published:** 2026-03-13

**Authors:** Anastasia V. Balakireva, Tatiana A. Karataeva, Michael Karampelias, Tatiana Yu Mitiouchkina, Viktor V. Morozov, Jan Macháček, Ekaterina S. Shakhova, Maxim M. Perfilov, Olga A. Belozerova, Sergey I. Kovalchuk, Kseniia A. Palkina, Nikola Drážná, Zuzana Vondráková, Karel Müller, Tetiana Kalachova, Aubin Fleiss, Josefina Patricia Fernandez-Moreno, Jose M. Alonso, Anna N. Stepanova, Liliia I. Fakhranurova, Nadezhda M. Markina, Dmitry A. Gorbachev, Evgenia N. Bugaeva, Galina M. Delnova, Vladimir V. Choob, Ilia V. Yampolsky, Jan Petrášek, Alexander S. Mishin, Karen S. Sarkisyan

**Affiliations:** 1 Planta LLC, Moscow, Russia; 2 Shemyakin-Ovchinnikov Institute of Bioorganic Chemistry, Russian Academy of Sciences, Moscow, Russia; 3 Institute of Experimental Botany of the Czech Academy of Sciences, Prague, Czechia; 4 Synthetic Biology Group, MRC Laboratory of Medical Sciences, London, UK; 5 Institute of Clinical Sciences, Faculty of Medicine, and Imperial College Centre for Engineering Biology, Imperial College London, London, UK; 6 Department of Plant and Microbial Biology, North Carolina State University, Raleigh, NC USA; 7 Departamento de Bioquímica y Biología Molecular, Campus Universitario de Rabanales y Campus de Excelencia Internacional Agroalimentario 3, Universidad de Córdoba, 14071 Cordoba, Spain; 8 Botanical Garden of Lomonosov Moscow State University, Moscow, Russia; 9 Pirogov Russian National Research Medical University, Moscow, Russia; 10 Light Bio Inc., Ketchum, ID USA

**Keywords:** Bioluminescence imaging, Biotic, Expression systems, Microbiology techniques

## Abstract

Jasmonic and salicylic acids are key hormones involved in plant responses to pests and pathogens. Existing fluorescence-based approaches to imaging plant defence hormones are constrained by the need for external illumination and by autofluorescence of plant tissues, while luminescence-based ones require exogenous substrates. Here, we use jasmonate- and salicylate-responsive promoters to engineer autoluminescent plants that report hormone signalling activity with up to a 53-fold contrast. Using consumer-grade cameras, we image reporter *Arabidopsis thaliana* and *Nicotiana benthamiana* plants throughout normal development and in response to pest and pathogen attacks, visualising local and systemic responses. Because the luminescence is self-sustained, these reporters enable non-invasive, substrate-free imaging of defence signalling over extended time courses without specialised equipment.

## Abstract (web summary)

Salicylic acid and jasmonic acid are two major hormones regulating plant response against pests and pathogens. Here, the authors configured the autoluminescence pathway to be conditional on activities of these hormones, achieving direct visualisation of plant defense responses.

## Introduction

Salicylic acid (SA) and jasmonic acid (JA) are plant hormones involved in regulating plant defence mechanisms and development. Salicylic acid primarily mediates response to biotrophic pathogens^1^, while jasmonic acid orchestrates defence responses against necrotrophic pathogens and herbivores^2^. Both hormones play essential roles in modulating stress responses, growth, and development. The relationship between salicylic and jasmonic acid pathways is mostly described as antagonistic^3^. This antagonism can be exploited by pathogens. For example, *Pseudomonas syringae* suppresses salicylic acid response by elevating the levels of jasmonic acid^4^, while whiteflies tilt the response towards salicylic acid^5^. The interplay of jasmonic and salicylic acids regulates local and systemic responses to pest attacks; these interactions are in turn affected by other hormones, including ethylene and auxin^6,7^.

How active are these hormones in different plant tissues in various ecological contexts, how local is their action, and how does it develop over time? A non-invasive imaging approach offering high spatial and temporal resolution could enable studies aimed at answering these questions. Existing approaches are generally limited to the use of fluorescent probes that require external illumination^8^, transcriptional luminescent reporters coupled with exogenous addition of luciferins^9^, or mass-spectrometry-based techniques that are typically invasive and rely on expensive equipment^10^.

**Fig. 1:**
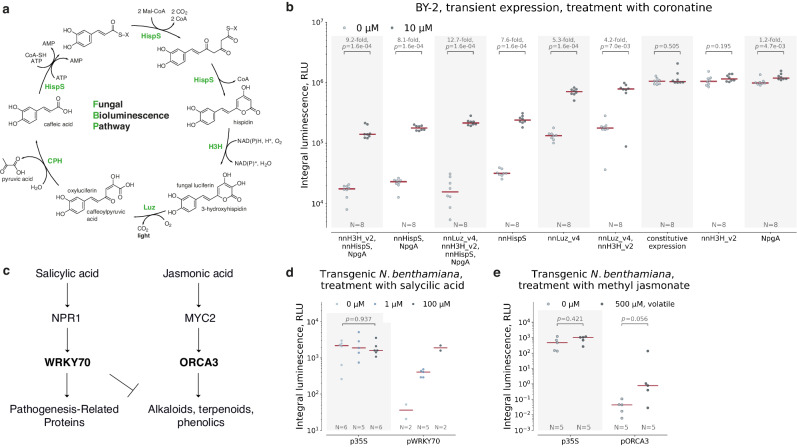
Prototyping the design of reporters based on the fungal bioluminescence pathway. **a** Biochemical reactions of the fungal bioluminescence pathway are catalysed by hispidin synthase HispS, hispidin-3-hydroxylase H3H, luciferase Luz, and putative caffeoyl pyruvate hydrolase CPH. For its activity, HispS requires a post-translational modification by a phosphopantetheinyl transferase, such as NpgA from *Aspergillus nidulans*. Fungal bioluminescence pathway proteins are coloured green. **b** Performance of various architectures of transcriptional reporters in tobacco BY-2 cell packs. Genes controlled by the jasmonate-responsive promoter pORCA3 are indicated on the horizontal axis. The rest of the genes were controlled by the constitutively active promoters: p35S-nnLuz-act2T; pCmYLCV-npgA-ATPaseT; pFMV-nnH3H-nosT; p35S-nnCPH-ocsT; p35S-nnHispS-ocsT. Concentrations of jasmonic acid agonist coronatine, that was used as an inducer, are specified in the legend. *N* = 8 technical replicates for each sample. The difference between mean values and *p*-values of post-hoc two-sided Mann–Whitney U tests corrected by the step-down method using Šidák adjustments are indicated above the brackets between the box plots. **c** Simplified presentation of signalling pathways induced by salicylic and jasmonic acids. WRKY70 promotes salicylic acid response, while inhibiting jasmonic acid signalling. ORCA3 is a transcription factor whose expression is activated by jasmonates. The ORCA3 promoter contains a jasmonate-responsive element that operates through interactions with transcription factors like AtMYC2. Inducibility of pORCA3-nnLuz (**d**, *N* = 5 biological replicates for each sample; the difference between mean values and *p*-values of post-hoc two-sided Mann–Whitney U tests corrected by the step-down method using Šidák adjustments are indicated above the brackets between the box plots) and pWRKY70-nnLuz (**e**, *N* = 2–5 biological replicates for each sample; the difference between mean values and *p*-values of post-hoc two-sided Mann–Whitney *U* tests corrected by the step-down method using Šidák adjustments are indicated above the brackets between the box plots) in transgenic *Nicotiana benthamiana*. The experiments were conducted on 1-week-old seedlings. The red line is the median, the coloured points represent individual data points. Source data are provided as a Source Data file.

In contrast, autoluminescence pathways produce the luminescence substrate biosynthetically in the studied organism^11–18^, potentially allowing for non-invasive imaging of molecular physiology. In the fungal bioluminescence pathway (Fig. 1a), caffeic acid, a molecule present in all land plants, is converted into luciferin^19–21^. The pathway can be reconstituted in plants by expressing five fungal genes: hispidin synthase HispS, a phosphopantetheinyl transferase such as NpgA from *Aspergillus nidulans*, hispidin-3-hydroxylase H3H, luciferase Luz, and caffeylpyruvate hydrolase CPH^13,16,22–25^. Here, we perform a proof-of-concept study, adapting the pathway to create transcription-based autoluminescence reporters that visualise hormone activities *in planta*.

## Results

### Identifying the optimal genetic construct architecture

We first tested what genetic architecture would produce optimal light emission without compromising the dynamic range. Previous studies pointed to HispS-catalysed reaction as the bottleneck of the pathway^23,24,26^, suggesting that inducible expression of hispidin synthase could provide tighter control over luminescence. However, we hypothesised that due to weak activity of hormone-sensitive promoters, insufficient levels of hispidin synthase might result in low signal-to-noise ratio, suggesting that another gene could be a better choice.

We tested different designs encoding bioluminescence pathway, using inducible promoters previously validated in our laboratory: jasmonic-acid-sensitive *Catharanthus roseus* pORCA3^27,28^ and cytokinin-sensitive promoter *Arabidopsis thaliana* pARR6^29^. In these designs, one or more genes of the pathway were controlled by pORCA3 or pARR6, while the remaining genes were expressed under strong constitutive promoters. In plant cell packs made of *Nicotiana tabacum* BY-2 cells^30^, luminescence increased in most reporter constructs upon induction (Fig. 1b, Supplementary Fig. 1). The HispS-NpgA-inducible design indeed showed highest activation and lowest background signal, in agreement with the previously reported data (Supplementary Fig. 1a,b)^23,24,26^. However, its luminescence in the induced state was dim; and in addition, transgenic plants expressing the bioluminescence enzymes but lacking hispidin synthase had measurable background level of luminescence (Supplementary Fig. 2), indicating a non-negligible levels of endogenous hispidin-like precursors, and disfavouring the HispS-based architecture. In contrast, Luz-inducible design demonstrated brighter luminescence, while showing a level of activation comparable to that of HispS-inducible design. We thus settled on the design where only the luciferase was driven by a hormone-sensitive promoter, while the rest of the genes were expressed constitutively.

### Validation of autoluminescent reporters in transient and stable assays

To construct a jasmonate reporter, we placed the *nnLuz_v4* gene^23^ under control of the promoter and terminator of *C. roseus ORCA3* gene^27^. For salicylic acid, we used the corresponding regulatory elements from *A. thaliana WRKY70* gene^29^ activated by salicylic acid and repressed by jasmonates (Supplementary Data 1)^31^. Specificity and limitations of these promoters were previously characterised in the literature (Supplementary Data 2).

Screening of our reporting constructs in BY-2 cell packs showed that pORCA3-inducible reporter responded to treatment with methyl jasmonate with an increase in luminescence (Supplementary Fig. 3). In the case of *pWRKY70*, we observed high luminescence in untreated samples, likely reflecting upregulation of *WRKY70* in response to *Agrobacterium* infiltration in our assay^32^ (Supplementary Fig. 3).

We continued evaluating the reporters in transgenic *Nicotiana benthamiana* and *A. thaliana* lines. We first created luciferin-producing master lines NB237 and AT8462, respectively, that constitutively expressed all bioluminescence genes, except luciferase (Supplementary Table 1, Supplementary Fig. 4). These lines were used to create plants with pORCA3-, pWRKY70- or p35S-driven expression of luciferase. Expression of key jasmonic and salicylic acid genes in hormone-sensing lines AT8463 (pWRKY70) and AT8464 (pORCA3) was not significantly activated compared to the parental Col-0 line (Supplementary Fig. 5). *N. benthamiana* plants stably expressing pWRKY70-inducible luciferase responded to treatment with salicylic acid in a concentration-dependent manner, showing up to 53-fold overall increase in light emission (Fig. 1d, the setup is shown at Supplementary Fig. 6). Accordingly, pORCA3 reporter plants showed an increase in luminescence upon treatment with methyl jasmonate (Fig. 1e). qPCR analysis of transcript levels of pWRKY70*-* and pORCA3-based reporters showed an expected increase in genes expression associated with salicylic and jasmonic acid-driven responses (Supplementary Fig. 7, Supplementary Table 2). We also assessed the specificity of the reporters by testing them against a panel of inducers, confirming that other physiological stimuli may influence their activation in vivo (Supplementary Figs. 8–10).

### Evaluation of reporters upon wounding and pathogen attacks

We then tested these plant lines in wounding experiments and upon pathogen attack. Wounding and infection with necrotrophic pathogens is known to raise the level of jasmonic acid (Supplementary Fig. 11). Infection with biotrophic pathogens, in turn, leads to the accumulation of salicylic acid^33^. In accordance with this, wounding of JA-reporting *N. benthamiana* plants induced luminescence locally within 2-3 hours (Supplementary Fig. 12 and 13, Supplementary Videos 1 and 2). Quick local induction of luminescence at infiltration sites was also observed in lines constitutively expressing the luciferase, likely reflecting activation of phenylpropanoid metabolism^16^. Analysis of leaves with LC-MS confirmed an increase in levels of jasmonic acid characteristic of the response to wounding (Supplementary Fig. 11).

**Fig. 2:**
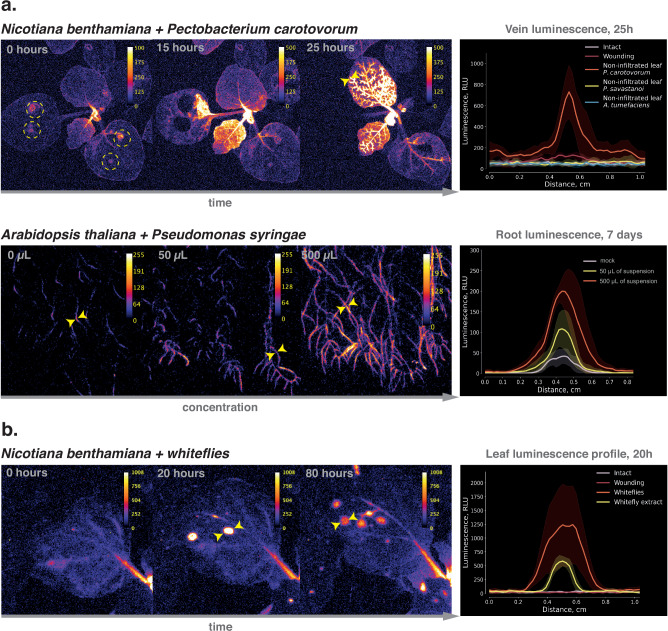
Performance of reporters of salicylic and jasmonic acids in transgenic Nicotiana benthamiana and Arabidopsis thaliana plants. **a** Pathogen attack, JA reporter—luminescent imaging of JA-reporting pORCA3-nnLuz-ORCA3_T expressing *N. benthamiana* (up) and *A. thaliana* (bottom) upon infection with *Pectobacterium carotovorum* (sites of infection are indicated by yellow circles with dashed lines) or *Pseudomonas syringae* pv. tomato DC3000, respectively. Pst50 and Pst500—50 µL or 500 µL of suspension in 50 mL of buffer, respectively. Plots indicate *N. benthamiana* non-infiltrated leaf veins brightness compared to tissues nearby at 25 hours post infection (up, *N* = 6) and *A. thaliana* root brightness treated with different concentrations of bacteria, 7 days post-treatment (bottom, *N* = 6–7 roots). Data are presented as mean values ± SD. **b** Pest attack, SA reporter—luminescent imaging of SA-reporting pWRKY70-nnLuz expressing-WRKY70_T plants infested with whiteflies. Plots indicate the brightness of leaves’ local responses to whitefly bites. Line profiles were calculated on areas indicated by yellow arrows. *N* = 3–7 local responses. Data are presented as mean values ± SD. Source data are provided as a Source Data file.

In turn, SA-reporting plants reacted to infiltration with *Agrobacterium tumefaciens* within 2.5 h, but no reaction was observed with the buffer (Supplementary Fig. 14, Supplementary Video 3). Upon infiltration with hemibiotrophic pathogen *Pseudomonas savastanoi*, JA-reporting plants responded within 2.5 h, primarily to wounding itself (Supplementary Fig. 14a). In contrast, luminescence of SA reporters increased ~15-fold specifically upon infiltration with the pathogen (Supplementary Fig. 14a), in accordance with salicylic acid response described in the literature^34,35^. In the case of a necrotrophic bacterium *Pectobacterium carotovorum*, we observed a striking increase in luminescence emitted from the vasculature of non-infiltrated, or systemic, leaves of JA-reporting plants at 12–14 h post-treatment (Fig. 2a, Supplementary Video 1, Supplementary Fig. 14a), also in accordance with existing data^2^. This vasculature signal likely visualises early stages of induced systemic resistance.

A similar behaviour was observed in *Arabidopsis thaliana* reporter lines. We incubated plants with *E. coli*, *A. tumefaciens*, and *P. syringae pv. tomato DC3000*, tracking their luminescence for seven days. The induction of the JA pathway by *P. syringae* is expected, since it produces coronatine—jasmonate mimic—to exploit the interplay between JA and SA pathways^36^. While JA-reporting plants showed no increase in luminescence upon treatment with *E. coli* or *A. tumefaciens*, they demonstrated a gradual dose-dependent response to *P. syringae* in the infected lateral roots during 7-day-long treatments (Fig. 2a, Supplementary Figs. 15 and 16).

In contrast, SA-reporting *Arabidopsis* plants did not respond to pathogenic *P. syringae*, showed minor response in the roots to high concentrations of *E. coli*, and responded to *A. tumefaciens*. During the first days of infection, *A. tumefaciens* reduced luminescence in the roots, and induced it in the shoots. After the first four days, pathogenesis advanced, resulting in halted development and chlorotic leaves (Supplementary Fig. 17).

### Autoluminescent reporters’ performance under pest attack

Taking advantage of the abundance of greenhouse whiteflies *Trialeurodes vaporariorum* in one of our facilities, we also aimed to monitor response of *N. benthamiana* plants to their attack in a seven-day experiment (Fig. 2a, Supplementary Figs. 12–14 and 18–20, Supplementary Videos 4–12). Previous studies showed that whitefly salivary protein effector Bt56 activates salicylic acid signalling, lowering the level of jasmonic acid and restraining plant response against the insect^5^. We released whiteflies onto reporter plants and imaged them for seven days (Supplementary Videos 4–12). No luminescence response was observed from jasmonate-reporting plants (Supplementary Fig. 13b). In contrast, salicylic acid reporter plants developed light-emitting spots, likely visualising local response to whitefly bites (Fig. 2b). The response started at the 15th hour post-treatment and ended ~135 h later. When we wounded leaves with needles dipped in the control buffer, we did not observe a response. But if the needles were dipped in homogenised whiteflies, wounding led to a notable response (Supplementary Fig. 14b), indicating specificity to whiteflies. On the sixth day of imaging, we registered an overall increase in brightness of plants bitten by whiteflies, potentially illustrating the development of a systemic acquired resistance triggered by the Bt56 effector^37,38^ (Supplementary Fig. 19). LC-MS analysis confirmed an increase in salicylic acid concentration in whitefly-bitten plants (Supplementary Fig. 20).

### Life-long imaging of autoluminescent reporters

We then performed long-term imaging of the normal development of reporter plants. We followed *N. benthamiana* development for over 7 weeks, starting from 4-leaf plants (Supplementary Video 13-15). The JA-sensing line was dim throughout the imaging experiment and became significantly brighter at flowering (Supplementary Fig. 21), demonstrating bright luminescence from floral primordia and the tube of the mature flower, whereas in limbs and sepals the brightness rapidly decreases after the flower opening, in agreement with existing data on elevated jasmonate activity in reproductive tissues^39^. Leaf veins of these plants emitted little light (Supplementary Fig. 21). In contrast, SA-sensing plants showed brighter luminescence from leaf vasculature and almost no light from flowers (Supplementary Fig. 21)^40^. The contrasting pattern of luminescence from leaf vasculature in two reporter lines may reflect the antagonistic relationship between salicylic acid and jasmonates. Control plants constitutively expressing bioluminescence genes (Supplementary Fig. 21) had evenly distributed luminescence emitted by leaves, flowers and stems.

## Discussion

The reporters described here allow non-invasive organism- and population-scale visualisation of molecular events. These reporter lines are based on previously used native plant promoters, which contain diverse motifs in their sequences and may have limited specificity: we recommend validating conclusions derived from imaging of these lines using orthogonal approaches. At the same time, the master lines we presented can be used to engineer other reporters, including those based on less complex, synthetic regulatory elements. To illustrate this possibility, we engineered a synthetic jasmonate reporter pJAZ1(x5)-nnLuz that showed a 50-fold increase in luminescence in response to treatment with jasmonate isoleucine (Supplementary Figs. 22 and 23a), as well as similar constructs based on SA-responsive elements (Supplementary Figs. 22 and 23b).

Although approaches based on autoluminescence have drawbacks, such as dependence of signal on metabolic activity of a tissue, they can provide insightful observations that may be further tested by other approaches. Ability to perform luminescence imaging without exogenous substrate and with inexpensive off-the-shelf consumer cameras enables studies of plant molecular physiology in the field, at scale, and in low-resource settings. We hope that the reporters described here will be useful in visualising defence responses of plants not only in the lab but also in their natural environment.

## Methods

### Design and assembly of genetic constructs

Coding sequences of bioluminescence genes were optimised for expression in *N. benthamiana* and ordered synthetically (Supplementary Data 1). Golden Gate assembly was performed in the T4 ligase buffer (Thermo Fisher) containing 10 U of T4 ligase, 20 U of either BsaI or BpiI (Thermo Fisher) and ~100 ng of each DNA part. Typically, Golden Gate reactions were performed according to ‘troubleshooting’ cycling conditions: 25 cycles (90 s at 37 °C, 180 s at 16 °C), then 5 min at 50 °C and 10 min at 80 °C. Oligonucleotides were generated in Evrogen.

Correct DNA assembly was typically confirmed by Sanger sequencing, and in some cases additionally by Nanopore or Illumina-based whole plasmid sequencing. DNA assembly and whole-plasmid sequencing were typically ordered from Cloning Facility (cloning.tech).

To generate stable *N. benthamiana* plants expressing either salicylic- or jasmonic-acid-responsive reporters, we first developed a “luciferase-less” masterline carrying genes necessary for fungal luminescence, except the luciferase gene. To obtain such masterline we assembled Level P plasmid pNK091 carrying (1) *nnCPH* gene under the control of 0.4 kb constitutive *35S* promoter from cauliflower mosaic virus with 5′ untranslated region of TMV omega virus and *ocs* terminator from *A. tumefaciens*; (2) wild-type version of *nnH3H* gene under the control of constitutive *FMV* promoter from figwort mosaic virus and *nopaline synthase* (*Nos*) terminator from *A. tumefaciens*; (3) *nnHispS* gene under the control of 0.4 kb constitutive *35S* promoter from cauliflower mosaic virus with 5′ untranslated region of TMV omega virus and *ocs* terminator from *A. tumefaciens*; (4) *NpgA* gene under the control of the constitutive *CmYLCV 9.11* promoter from Cestrum yellow leaf curling virus and *ATPase* terminator from *Solanum lycopersicum*; (5) kanamycin resistance cassette driven by *pNos* promoter and *ocs* terminator from *A. tumefaciens*.

The master line NB237 was then further transformed with plasmids encoding phytohormone-sensing luciferase-carrying transcription units. To obtain phytohormone-sensing plants, masterline was transformed with the Level M plasmid carrying hygromycin resistance cassette driven by *pNos* promoter and *ocs* terminator from *A. tumefaciens* and *nnLuz_v4*^23^ under the control of *WRKY70* promoter (2060 bp upstream of *A. thaliana WRKY70* start codon, GenBank: LR782544.1) and terminator (500 bp downstream *A. thaliana WRKY70* stop codon, GenBank: LR782544.1) in the case of salicylic acid reporter (plasmid ID: M7691) or under the control of *ORCA3* promoter (826 bp upstream *C.roseus ORCA3* start codon, GeneBank: AJ251250.1) or terminator (385 bp downstream *C.roseus ORCA3* stop codon, GeneBank: AJ251250.1)—in the case of jasmonic acid reporter (plasmid ID: M7138). In control samples *nnLuz_v4* gene was placed under the constitutive 0.4 kbp *35S* promoter and *AtAct2* terminator (plasmid ID: pNK3558).

### Building JAZ1(x5) and PR1(x3) synthetic promoters

For building the hormone-inducible synthetic promoters JAZ1(x5) and PR1(x3), genomic regions harbouring potential hormone-specific transcription factor binding sites were used and multimerized in tandem. For the JAZ1(x5) promoter, five copies of an 86 nucleotide-long sequence located in the second exon of the 5’UTR of the first cDNA isoform of Arabidopsis *JAZ1* gene (*At1g19180.1*), stretching from nucleotides −352 to −267 upstream of the translation start codon, were used (Dr. Roberto Solano, personal communication) (Supplementary Fig. 22a). This sequence corresponds to the putative core promoter of the third *JAZ1* cDNA isoform (*At1g19180.3*) and a more distal promoter section of the second *JAZ1* cDNA isoform (*At1g19180.2*). In this work, a tandem of five copies of this sequence were followed by downstream minimal 35S promoter and *nnLuz* reporter preceded by the 5’UTR of Arabidopsis *RUBISCO SMALL SUBUNIT 2B (RbcS2B)*. For the PR1(x3) promoter, three copies of a 192-194 nucleotide-long promoter fragment located at nucleotides −851 to −657 upstream of the start codon of *At2g14610.1* were utilised^41^.

The strategy used for their assembly and cloning utilised the GoldenBraid (GB) multicloning technology^42,43^ and GB-like 4 nt codes to assemble multiple repeats into a single promoter in one cloning step^43^. For *JAZ1*, the DNA sequence was synthesised commercially as part of multiple IDT gBlocks, whereas for *PR1* promoter, the fragment of interest was directly amplified from Arabidopsis genomic DNA^44^. Due to the repetitiveness of the desired *JAZ1* sequence, the synthesis of the complete JAZ1(x5) promoter was not possible, so each repeat was synthetised individually (as part of bigger gBlocks also containing unrelated sequences for other lab projects) and flanked by a pair of variable sequences corresponding to existing lab primers that could then be used for their individual amplification (Supplementary Table 3) and making use of the GB 4 nt grammar (the 3’ code for B1, B3, B4 and B4 GB bricks: [CCAT], [AGCC], [TTCG] and [GCTT], respectively) to guide the assembly of the five repeats simultaneously into pUPD2 GB entry vector (Supplementary Fig. 22a). The final promoter JAZ1(x5) was flanked by the distal-proximal promoter GB grammar A1-A2 [GGAG-TCCC] (Supplementary Fig. 22a). For the *PR1* promoter, the desired sequence was amplified with two sets of oligo pairs designed to remove the GB type IIS enzyme restriction site present within the sequence, and subcloning the two amplified fragments into a single pUPD2 entry vector (Supplementary Table 3, Supplementary Fig. 22b). In a second round of amplifications using this domesticated clone as a template, the fragment was re-amplified separately in three reactions with three sets of oligo pairs following a similar approach as for JAZ1, in which each repeat was separated by a 4 GB-like nt code (GBL1 [CAGT] and GBL4 [AGCA])^43^, and the final PR1(x3) promoter was flanked by the distal-proximal promoter GB grammar A1-A2 [GGAG-TCCC] (Supplementary Fig. 22b).

Level 1 genetic constructs carrying *pJAZ1(x5)* (*pAS13_pUPD2_JAZ1(x5)*) and *pPR1* (*pAS11_pUPD2_PR1(x3)*) were assembled with core minimal *35S* promoter and the 5’ UTR of *RbcS2B* and the *Act2* terminator. Other hormone-reporting constructs were assembled using standard MoClo parts: *pJOG025 - p(At)EDS1*, *pJOG026 - p(At)PAD4*, *pJOG466 - p(At)SAG101*, *pJOG010 - p(At)PR1* and contained all standard regulatory elements.

### Transformation of *Agrobacterium tumefaciens*

Plasmids were transformed into competent cells of *A. tumefaciens* AGL0^45^, and clones were selected on LB (Luria-Bertani) agar plates containing 50 mg/L of rifampicin and an additional antibiotic, depending on the plasmid used for transformation (200 mg L^−1^ of carbenicillin, 50 mg mL^−1^ of kanamycin or 100 mg mL^−1^ spectinomycin). Individual colonies were then inoculated into 10 mL of LB medium containing the same concentration of antibiotics. After overnight incubation at 28 °C with shaking at 220 rpm, cultures were centrifuged at 2900 *g*, resuspended in 25% glycerol and stored as glycerol stocks at −80 °C.

### Growth conditions for *Arabidopsis thaliana*

Seeds were surface sterilised with 70% ethanol for 15 min and washed twice with 96% ethanol. Seeds were dried in a laminar flow and then sown on the surface of solid (1% plant agar) half-strength MS medium with 1% sucrose. Sown seeds were vernalized for 2 days at 4 °C and then transferred to grow vertically at 22 °C.

### Validation of reporters in plant cell packs based on *Nicotiana tabacum* BY-2 cell culture

Tobacco BY-2 cell culture was grown in BY-2 medium (Murashige and Skoog (MS) with 0.2 mg L^−1^ 2,4-dichlorophenoxyacetic acid, 200 mg L^−1^ KH_2_PO_4_, 1 mg L^−1^ thiamine, 100 mg L^−1^ myo-inositol and 30 g L^−1^ sucrose) at 27 °C by shaking at 130 rpm in darkness, with 2 mL of 1-week-old culture being transferred into fresh 200 mL of BY-2 medium every week^46^.

Transformations of BY-2 cell packs were made according to a protocol adapted from a previous study^47^and included the following steps. One-week-old BY-2 culture was pelleted in black 96-well plates to create cell packs that were infiltrated with a mixture of several agrobacterial strains containing binary vectors. One of the strains encoded silencing inhibitor P19 (OD_600_0.2), and other encoded bioluminescence genes and a reporter (*ARR6-, WRKY70-, ORCA3-nnLuz_v4* variants) gene (OD_600_0.5). Comparison of reporters was done by co-infiltrating BY-2 cell packs with agrobacteria individually encoding bioluminescence enzymes.

Salicylic acid and jasmonic acid reporter functionality was tested in BY-2 cell packs. In the case of *pWRKY70-nnLuz_v4-WRKY70_T*, the cell packs were treated with 100 μM salicylic acid solution. The 100 µL of salicylic acid solution was applied on the cell packs for 30 min, the excess of the solution was removed with centrifugation for 1 min, 500 *g*. In the case of *pORCA3-nnLuz_v4-ORCA3_T*, the cell packs were treated with methyl jasmonate emitted from a surface of 500 μM solution for the duration of the experiment (12–24 h) placed in between the rows of 96-well plates.

After the treatments, the plates were incubated at 80% humidity at 22 °C and immediately imaged for 48 h using Sony Alpha ILCE-7M3 camera and 35-mm T1.5 ED AS UMC VDSLR lens (Samyang, ~*f*/1.4) with an exposure of 5–30 s and ISO 400, 3200, and 20,000.

### Agrobacterium-mediated transformation of *Nicotiana benthamiana*

*A. tumefaciens* strains AGL0 carrying plasmid pNK091 (for creation of a masterline) or plasmids M7138, M7691, pNK3558 (for the development of reporter lines) were grown in flasks on a shaker overnight at 28 °C in LB medium supplemented with 25 mg L^−1^ rifampicin and 50 mg L^−1^ kanamycin or hygromycin, respectively. Bacterial cultures were diluted in liquid MS medium to an optical density of 0.6 at 600 nm. Leaf explants used for transformation experiments were cut from 2-week-old *N. benthamiana* plants and incubated with bacterial culture for 20 min. Leaf explants were then placed onto filter paper overlaid on MS medium (MS salts, MS vitamins, 30 g L^−1^ sucrose and 8 g L^−1^ agar, pH 5.8) supplemented with 1 mg L^−1^ 6-benzylaminopurine and 0.1 mg L^−1^ indolyl acetic acid. Two days after inoculation, explants were transferred to the same medium supplemented with 500 mg L^−1^ cefotaxime and 75 mg L^−1^ kanamycin or hygromycin. Regeneration shoots were cut and grown on MS medium with antibiotics.

### Transformation of *Arabidopsis thaliana*

*Arabidopsis thaliana* (ecotype Columbia 0) was transformed with floral dip^48^ with a binary vector carrying the transcriptional units for the ectopic production of the fungal luciferin, and the recycling of the luciferin’s oxidation product (caffeoylpyruvic acid). After four generations of antibiotic resistance (kanamycin), homozygous plants were transformed with a binary vector with a transcriptional unit regulating the expression of the fungal luciferase by the *ORCA3* or *WRKY70* promoters and corresponding terminators. Transgenic plants were selected for resistance to hygromycin, and bioluminescence was captured by the Sapphire (Azzure) bioimager (exposure time was 2 min). Second-generation, homozygous plants were used further for experiments.

### Validation of phytohormone reporters in transgenic *Nicotiana benthamiana*

All experiments were carried out on T1 plants selected by their resistance to antibiotic and by their ability to glow at two developmental stages: 1-week-old seedlings grown in vitro in Petri dishes, and 3-4-week-old plants grown in peat tablets. In all experiments, three stable lines of *N. benthamiana* were used: they carried transcription units of fungal bioluminescence genes: pNos-KanR-ocsT | p35S-nnHispS-ocsT | pCmYLCV-npgA-ATPT | p35S-nnCPH-ocsT | pFMV-nnH3H_wt-NosT – and either salicylic acid reporter pWRKY70-nnLuz_v4-WRKY70_T, jasmonic acid reporter pORCA3-nnLuz_v4-ORCA3_T, or control p35S_0.4kbp-nnLuz_v4-Act2. For each experiment, we used 3-5 plants as replicates.

### Wounding

To induce jasmonic acid signalling, we damaged the stems of plants grown in vitro or the leaves of the plants by cutting them with scissors.

### Salicylic acid and methyl jasmonate treatment

To induce salicylic acid signalling, we sprayed the plants with 100 μM salicylic acid solution and imaged the experiment for 48 h. To induce jasmonate signalling, we treated the plants with methyl jasmonate emitted from a surface of a cotton wool ball soaked in 5 mM solution, placed next to the plants, and imaged the experiment for 48 h.

### Infection with bacteria

We infected transgenic plants with bacteria using different infection strategies. For the experiments, we used a hemibiotrophic pathogen *Pseudomonas savastanoi* (a pathovar of *Pseudomonas syringae)* and necrotrophic bacteria *Pectobacterium carotovorum*. To infect the plants, we first damaged the stems with a needle and then applied the pathogens in wounded areas. In parallel, the effect of the *A. tumefaciens* strain AGL0 infection was studied. A 10 mM MgSO_4_, 0.01% Silwet L-77 solution in which bacteria were resuspended was used as a control. The OD_600_ of bacteria was 0.2. The imaging duration was 48 hours.

### Whitefly infestation

The greenhouse whitefly *Trialeurodes vaporariorum* was used in the experiments. A total of 100 whiteflies per plant was applied on assayed *Nicotiana* lines. These plants were covered with a glass fish tank to trap the insects. To imitate the whitefly bite, we pricked the leaves with sterile needles dipped in either 0.1 M phosphate buffer pH 7.0 (mock) or the whitefly extract. The extract was prepared by homogenising 50 whiteflies in 2 mL of the 0.1 M phosphate buffer pH 7.0. The imaging duration was 48 h.

### Reporter specificity test

To test the specificity of the *N. benthamiana* reporters, detached leaves of 4-week-old plants were used. Treatment with the hormones included water solutions of abscisic acid (100 μM, stock in ethanol), coronatine (10 μM, stock in DMSO), 1-naphthaleneacetic acid (NAA, 100 μM, stock in ethanol), thidiazuron (100 μM, stock in DMSO), epibrassinolide (10 μM, stock in ethanol), gibberellin GA₃ (10 μM, stock in ethanol), the synthetic strigolactone GR24 (10 μM, stock in DMSO), and 1-aminocyclopropane-1-carboxylic acid (ACC, 100 μM), along with positive controls, salicylic acid (SA, 100 μM, stock in ethanol) and jasmonate-isoleucine (JA-Ile, 1 mM, stock in DMSO).

### UVB treatment

The plants were exposed to 312 nm UV light (Vilber Lourmat) for 30 min.

### Salt treatment

The plants were watered with 200 mM solution of NaCl until the liquid was pouring from the pots. Water was used as a mock.

### Luminescent imaging

The plants were imaged for 48 h using a Sony Alpha ILCE-7M3 camera and 35-mm T1.5 ED AS UMC VDSLR lens (Samyang, ~f/1.4) with an exposure 30 s and ISO 400, 3200, and 20,000. We typically imaged plants every 30 min.

### Long-term time-lapse luminescent imaging of phytohormone-sensing plants

Long-term time-lapse, luminescent imaging of hormone-sensing plants was performed for 1.5 months using Sony Alpha ILCE-7M3 camera and 35-mm T1.5 ED AS UMC VDSLR lens (Samyang, ~*f*/1.4) with an exposure of 5–30 s and ISO 400, 3,200, and 20,000. The snapshots were captured every 30 min.

Processing of images was performed using FiJi ImageJ distribution (version 1.53t) and custom Python scripts. For luminescence quantification, mean values in the region of interest after background subtraction were used. Background subtraction was performed using the following formula:

1 $${{{\rm{Signal}}}}={{{\rm{Signal}}}_{raw}}-{{{\rm{Background}}}_{mean}}$$

### Treatments of transgenic *Arabidopsis thaliana* reporters with bacteria

The bacteria used in this study were *E. coli* (XL1 blue), *A. tumefaciens* (GV2260), and *P. syringae* pv. tomato DC3000. Single colonies grown on solid LB medium were inoculated into 1.5 mL of liquid LB, and the cultures were incubated in a shaker at 28 °C for *A. tumefaciens* and *P. syringae* and at 37 °C for *E. coli*. After 20 h of incubation, bacterial cultures were centrifuged at 1500 g for 5 min, LB was discarded, and pelleted bacteria were resuspended in autoclaved distilled water to O.D. 0.5. The infection medium was set in square plates with 50 mL of half-strength MS salts and 1% plant agar inoculated with 50 or 500 µL of bacterial suspensions (O.D. 1) while warm and liquid. Six-day-old seedlings grown vertically on half-strength MS salts with 1% agar were transferred to plates with the same medium, inoculated as mentioned above. One plate was free of bacteria and served as a control.

### Treatments of transgenic *Arabidopsis thaliana* reporters with phytohormones

To test the responsiveness and specificity of the SA (pWRKY70) and JA (pORCA) reporters in *A. thaliana*, 6-day-old seedlings vertically grown on half-strength MS salts with 1% agar and 1% sugar, were transferred on the same medium supplemented with methyl jasmonate (MeJa, 5 μM and 500 nM), salicylic acid (5 μM and 500 nM)1-naphthaleneacetic acid (NAA, 500 nM), indole-3-acetic acid (IAA, 500 nM), 1-aminocyclopropane-1-carboxylic acid (ACC, 1 μM), 6-benzylaminopurine (BAP, 500 nM), aminoethoxyvinylglycine (AVG, 1 μM), and trans-zeatin (500 nM) added as DMSO solutions to a final DMSO concentration 0.0001% v/v. The response in bioluminescence was recorded with a commercial camera (see imaging of *A. thaliana*) in the four subsequences starting 1 day after the day of treatment.

### RT-qPCR experiments

To assess the expression levels of the luciferase gene and pathogen-related genes, 10-day-old seedlings of *A. thaliana* were used, while for similar experiments in *N. benthamiana,* the leaves of 4-week-old plants were used. For each experiment, three biological and three technical replicates were used. The *N. benthamiana* RNA was extracted using Evrogen ExtractRNA BC032. cDNA was generated using Evrogen Mint-2 cDNA synthesis kit SK005, RT-qPCR was performed utilising Evrogen 5X qPCRmix-HS SYBR PK147S. *A. thaliana* RNA was extracted using the RNeasy by Qiagen. Primers are listed in Supplementary Tables 2 and 3.

### Imaging of *Arabidopsis thaliana*

Plants grown on media with bacteria were photographed on the indicated days (d.a.t., days after transfer) with a Samyang 35 mm f/1.4 AS UMC lens attached to a Nikon Z6 II camera body. Imaging included a photo in a well-lit condition (*f*/1.4, ISO 200, 1/160 s) and a photo in complete darkness (*f*/1.4, ISO 51200, 60 s). Plates with plants were placed horizontally for less than 2 min while imaging. Image processing by ImageJ software includes isolation of the green channel, denoising by outliers removal, and intensity increase.

### Quantification of bioluminescence in *Arabidopsis thaliana* reporter lines

The well-lit photo of each day was used to generate regions of interest in Fiji software for each root. Eight roots were used for each treatment. These ROIs were mapped on the green channel of the 16-bit TIFF files, which were generated from the raw imaging files from the 4 days of imaging in darkness. Intensity measurements were taken with Fiji, and the values were used to generate statistical outputs.

### Data presentation and statistical analysis

Most of the data are plotted as medians with coloured individual data points using Seaborn (https://seaborn.pydata.org/, ver. 0.13.2) and Matplotlib (https://matplotlib.org/, ver. 3.8.0) packages, using Python version 3.10.12. Pairwise post-hoc two-sided Mann–Whitney U or Welch’s two-sample *t*-test (Scikit-posthocs package^49^, version 0.10.0) were computed. Sample numbers (*N*) are reported in the figures.

### Chemicals for LC-MS experiments

The analytical standards of jasmonic acid, salicylic acid were purchased from Sigma-Aldrich® (≥98.0), hispidin was chemically synthesised and tested for purity in house (>95.0). A standard solution of two components was prepared in 50% acetonitrile. HPLC-grade acetonitrile was purchased from J.T.Baker. Deionized water was obtained from a Milli-Q System (USA) and acetic acid was purchased from Sigma-Aldrich (≥98.0).

### Sample preparation for LC-MS

The extraction and quantification procedure was adapted from our previously described protocol^24^. In brief, fresh leaves were homogenised in liquid nitrogen and lyophilised. Extracts were obtained from 25 mg of dry plant biomass using 1 mL of 70% methanol, filtered through 0.45 μm GF/PVDF (Phenex) filter and lyophilised in miVac machine. Dry residues were reconstituted in 100 µL of 70% methanol by vortexing and transferred for LC-MS analysis.

### LC-MS analysis

LC-MS analysis was carried out on an Ultimate 3000 RSLCnano HPLC system connected to a QExactive Plus mass spectrometer (Thermo Fisher Scientific). Samples were separated on a Gemini C18 3 μm NX LC column 100*2.1 mm (Phenomenex) at 200 µL/min flow rate. Separation was done by a linear gradient of 90% acetonitrile in water, 10 mM ammonium formate, 0.1% formic acid (Buffer B) in 99.9% H_2_O, 10 mM ammonium formate, 0.1% formic acid (Buffer A): 1% B at 0 min, 50% B at 3 min, 99% B at 8 min, followed by 3 min wash at 99% B and 2 min equilibration at 1% B before the next run. UV data were collected at 220 nm. MS1 spectra were collected in negative-ion mode at 30 K Orbitrap resolution with 100–1000 a.e.m mass range. MS2 spectra were collected at 15 K resolution. Fragmentation was performed by HCD with stepped CE of 25%, 30% and 35%. Raw data were collected and processed using Thermo Xcalibur Qual 4.0.27.19 (Thermo Fisher Scientific) software. The MS peaks were extracted at a mass tolerance of 5 ppm. For compound quantification, the corresponding peak area for each sample was used. Absolute concentrations were determined using calibration curves prepared from the corresponding standards (0.1 − 10 μg/mL) and expressed as nmol·g⁻¹ after conversion using molecular weights and normalisation to 25 mg dry weight. Data shown are means of three biological replicates.

### Reporting summary

Further information on research design is available in the Nature Portfolio Reporting Summary linked to this article.

## Supplementary information

Supplementary Information

Peer Review file

Description of Additional Supplementary Files

Supplementary Data 1

Supplementary Data 2

Supplementary Movie 1

Supplementary Movie 2

Supplementary Movie 3

Supplementary Movie 4

Supplementary Movie 5

Supplementary Movie 6

Supplementary Movie 7

Supplementary Movie 8

Supplementary Movie 9

Supplementary Movie 10

Supplementary Movie 11

Supplementary Movie 12

Supplementary Movie 13

Supplementary Movie 14

Supplementary Movie 15

Reporting Summary

## Source data

Source Data

## Data Availability

The plasmids used in this study are available for non-commercial use through Addgene [https://www.addgene.org/browse/article/28247826/]. Plant lines will be made available under an MTA upon request. Metabolomics data are available at MetaboLights repository^50^ under accession MTBLS13465. Raw imaging data is available at Figshare [10.6084/m9.figshare.30911858]. Source data are provided with this paper.

## Code availability

Python code for processing and plotting data is available at Github [https://github.com/Perfus/BL_plant_hormones].
