# Supplementary material for: Non-invasive imaging of defence responses in plants
Source: Nat Commun. 2026 Mar 13;17:6393. doi: 10.1038/s41467-026-70075-1 (PMC13376918; doi:10.1038/s41467-026-70075-1)
Supplement: Supplementary file 1 — Supplementary Information [file 41467_2026_70075_MOESM1_ESM.pdf]

Non-invasive imaging of defence responses in plants

Balakireva *et al.*

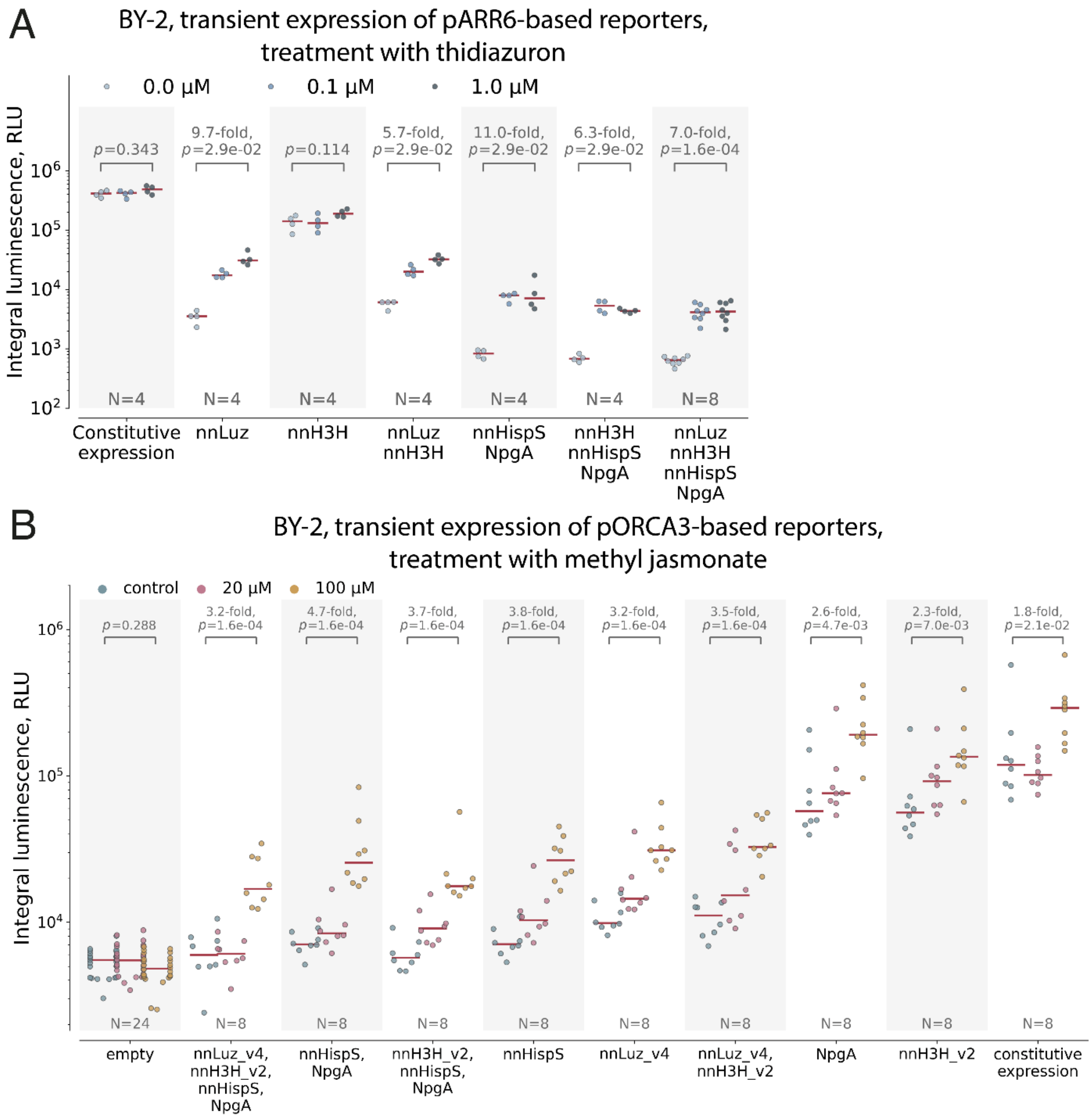

Supplementary Figure 1. Selection of a fungal bioluminescence gene as a reporter of phytohormone activity upon treatment with the inducer in BY-2 cell packs. A. Treatment of BY-2 cell packs expressing fungal bioluminescence genes put under *pARR6* with 0.1 µM and 1 µM thidiazuron solution. The samples were treated for 30 minutes before centrifugation. The red line is the median, the coloured points represent individual data points. The difference between mean values and *p*-values of post-hoc two-sided Mann–Whitney U tests are indicated above the brackets between the box plots. N = 4-8 plant cell packs. B. Treatment of BY-2 cell packs expressing fungal bioluminescence genes put under *pORCA3* with volatile methyl jasmonate throughout the experiment for 48 hours. The red line is the median, the coloured points represent individual data points. The difference between mean values and *p*-values of post-hoc two-sided Mann–Whitney U tests are indicated above the brackets between the box plots. N = 8-24 plant cell packs. c. Source data are provided as a Source Data file.

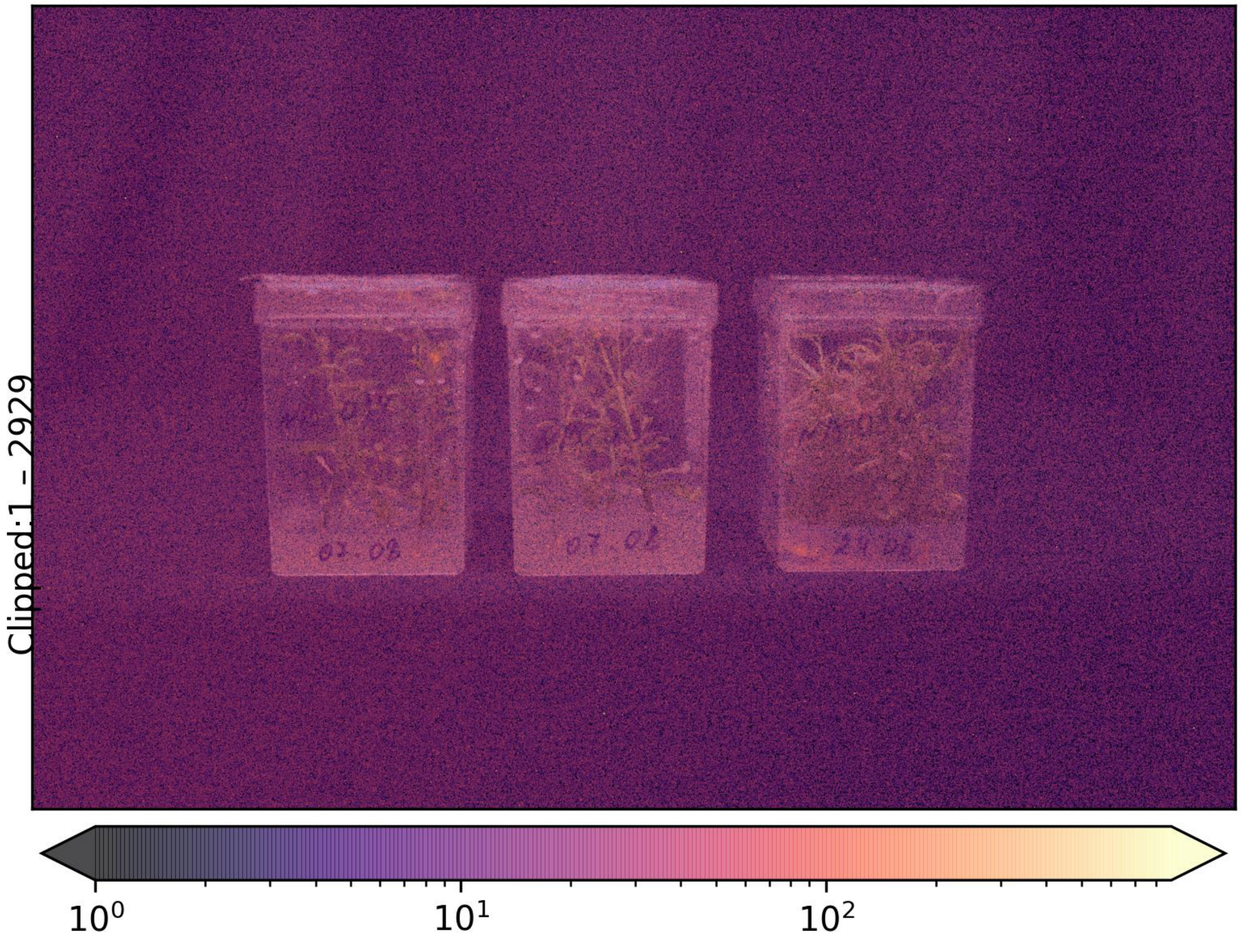

Supplementary Figure 2. Luminescence emitted by the hispidin synthase-lacking *Nicotiana benthamiana in vitro*-grown plants. Pseudocoloured luminescence image (Sony Alpha, ISO 20000, exposure 30 sec), overlaid onto a photo in ambient light.

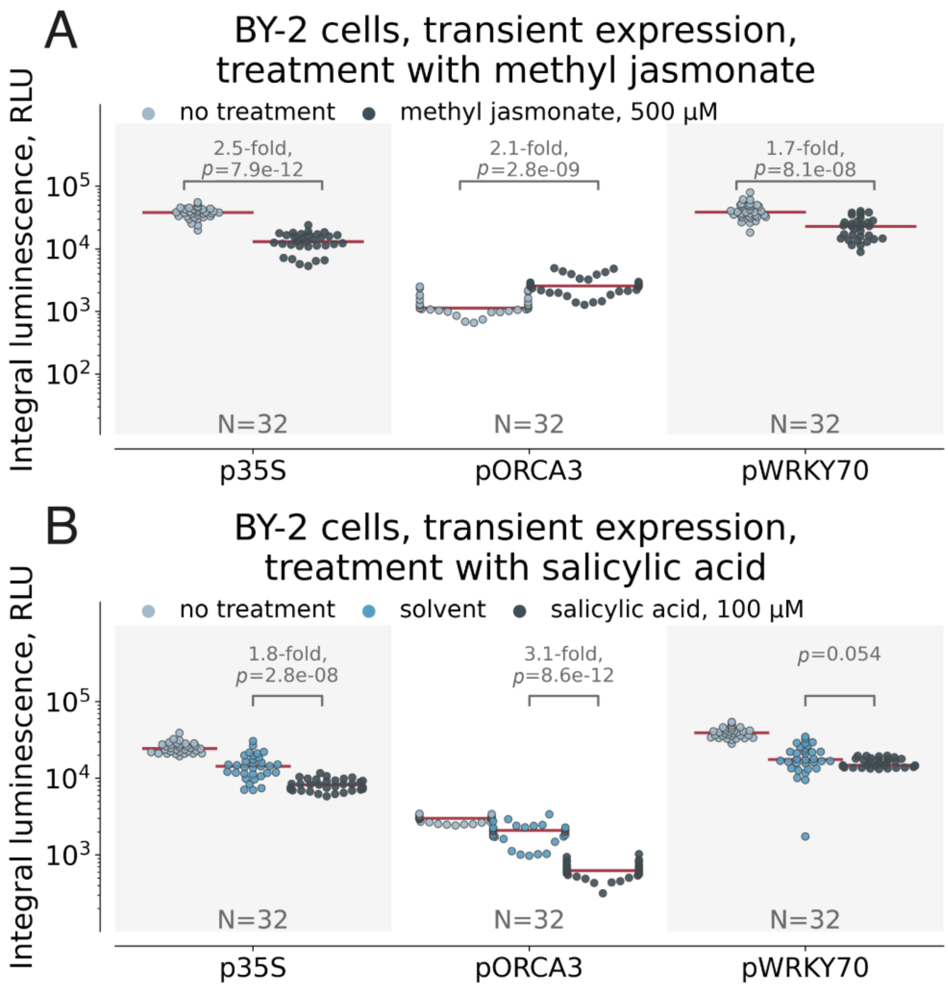

Supplementary Figure 3. Activation of jasmonic and salicylic acid reporters in BY-2. A. Treatment of pORCA3-, pWRKY70- and p35S-nnLuz_v4-expressing BY-2 cells with volatile methyl jasmonate throughout the experiment for 48 hours. The red line is the median, the coloured points represent individual data points. The difference between mean values and *p*-values of post-hoc two-sided Mann–Whitney U tests are indicated above the brackets between the box plots. N = 32 plant cell packs. Pairwise post-hoc two-sided Mann–Whitney U tests with *p* values corrected by the step-down method using Šidák adjustments were computed. B. Treatment of pORCA3-, pWRKY70- and p35S-nnLuz_v4-expressing BY-2 cells with 100 µM salicylic acid for 30 minutes prior to centrifugation. The red line is the median, the coloured points represent individual data points. The difference between mean values and *p*-values of post-hoc two-sided Mann–Whitney U tests are indicated above the brackets between the box plots. N = 32 plant cell packs. Pairwise post-hoc two-sided Mann–Whitney U tests with *p* values corrected by the step-down method using Šidák adjustments were computed. Source data are provided as a Source Data file.

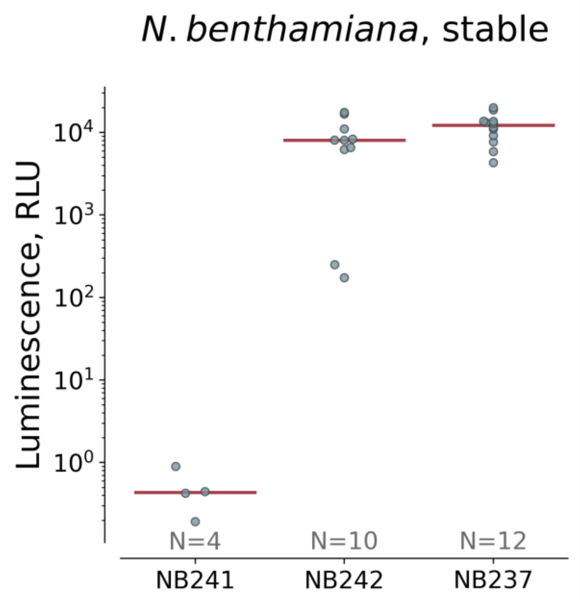

Supplementary Figure 4. Luminescence upon infiltration of leaves of independent luciferase-less *Nicotiana benthamiana* lines with agrobacteria encoding *p35S-nnLuz_v4*. NB237 was chosen as the masterline for development of other lines used in the project. The red line is the median, the coloured points represent individual data points. N = 4-12 plant cell packs. Pairwise post-hoc two-sided Mann–Whitney U tests with *p* values corrected by the step-down method using Šidák adjustments were computed. Source data are provided as a Source Data file.

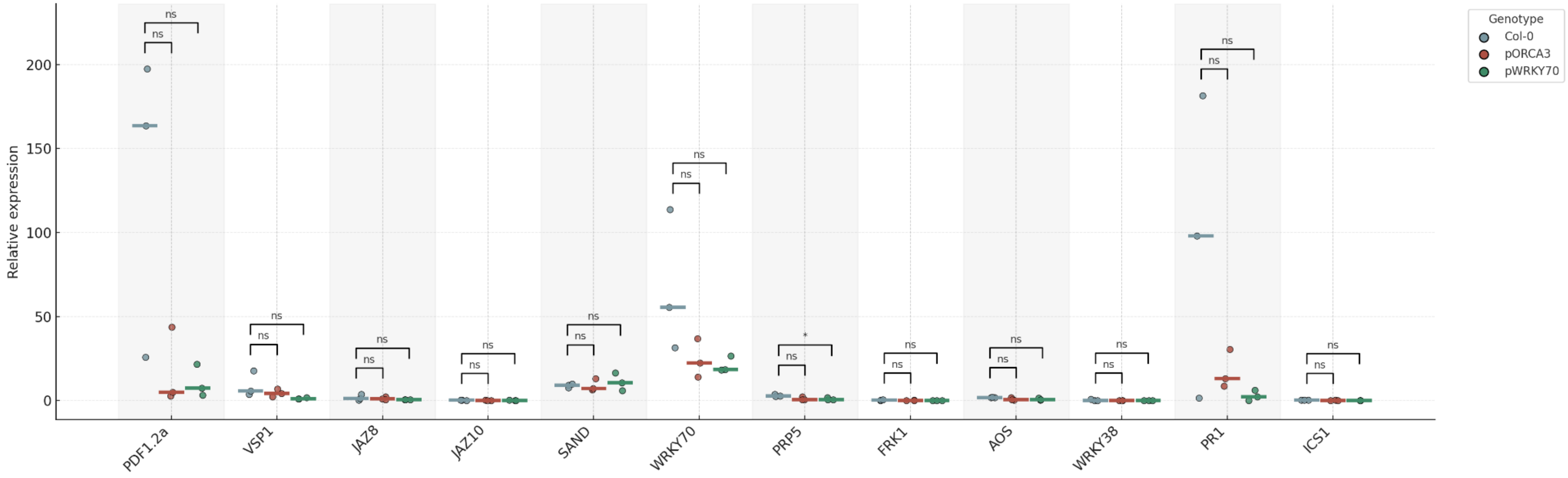

Supplementary Figure 5. The result of qPCR of pathogen-related genes in *Arabidopsis thaliana* Columbia-0 and transgenic *A. thaliana* expressing salicylic acid (*pWKRY70-nnLuz-WRKY70_T*)- or jasmonic acid (*pORCA3-nnLuz-ORCA3_T*) reporters. Genes tested: *Plant Defensin 1.2a* (*PDF1.2a*), *Vegetative Storage Protein 1* (*VSP1*), *Jasmonate-ZIM-domain protein 8* (*JAZ8*), *Jasmonate-ZIM-domain protein 10* (*JAZ10*), *SAND family protein* (*SAND*), *WRKY DNA-binding protein 70* (*WRKY70*), *Pathogenesis-Related protein 5* (*PRP5*), *Flg22-induced Receptor-like Kinase 1* (*FRK1*), *Allene Oxide Synthase* (*AOS*), *WRKY DNA-binding protein 38* (*WRKY38*), *Pathogenesis-Related protein 1* (*PR1*), *Isochorismate Synthase 1* (*ICS1*). *p*-values were calculated with Welch's two-sample *t*-test. Source data are provided as a Source Data file.

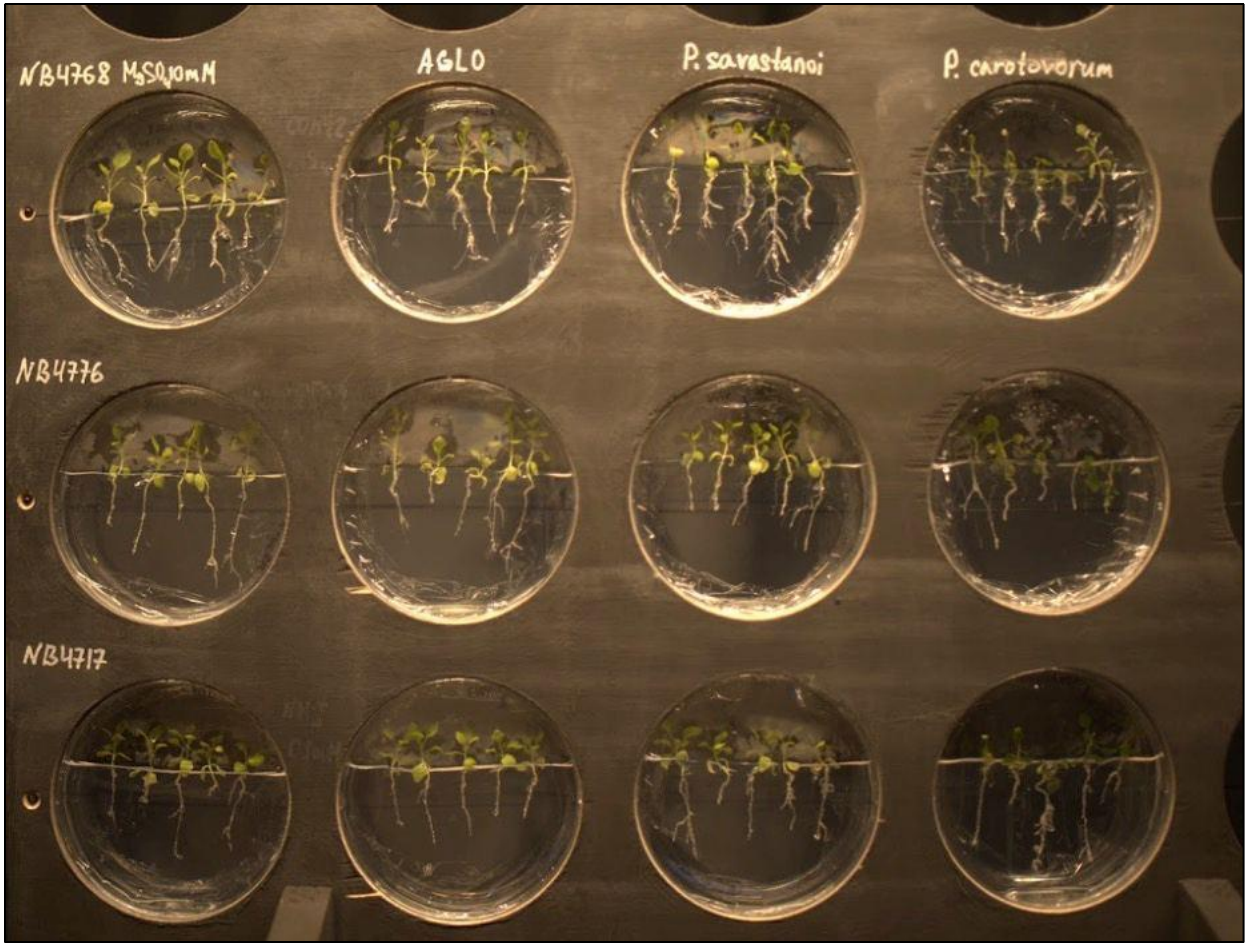

Supplementary Figure 6. A custom wooden setup used to image pathogen infection in plant seedlings.

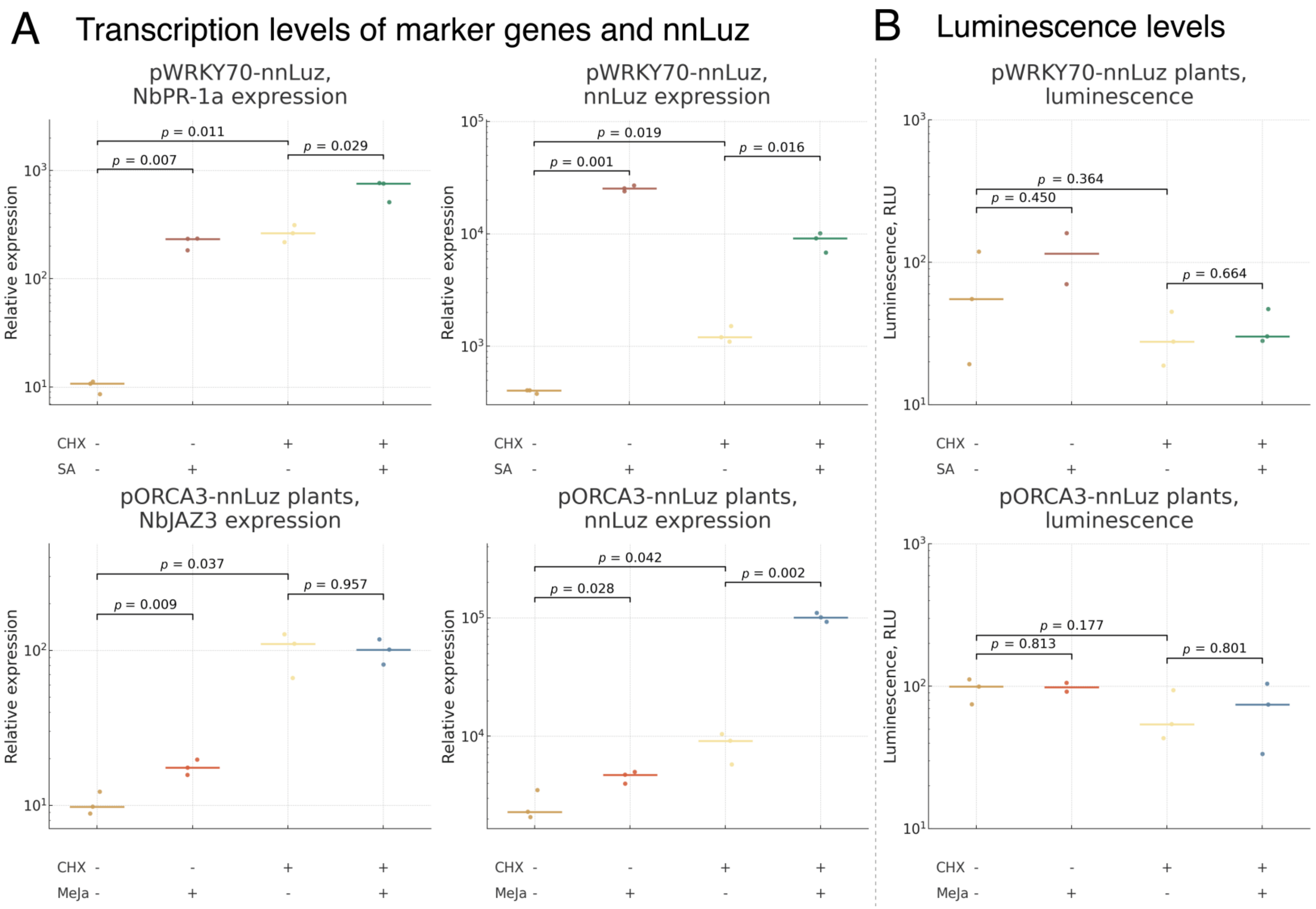

Supplementary Figure 7. Assessment of transcription and luminescence levels when reporters were induced with salicylic acid (100 µM) or methyl jasmonate (500 µM volatile) in the presence of protein translation inhibitor cycloheximide. A.) Transcription levels of marker genes for salicylic (NbPR-1a) and jasmonic acid (NbJAZ3) and the luciferase. Dots represent mean values out of 3 biological replicates. *p*-values were calculated with Welch's two-sample *t*-test, comparing the integral luminescence of the control (Mock) against each other treatment within the same promoter. B. Luminescence output of the corresponding samples. Leaves of 4-week-old plants were used. The hormones were applied once at the start of the experiment, the imaging lasted for 48 hours. Source data are provided as a Source Data file.

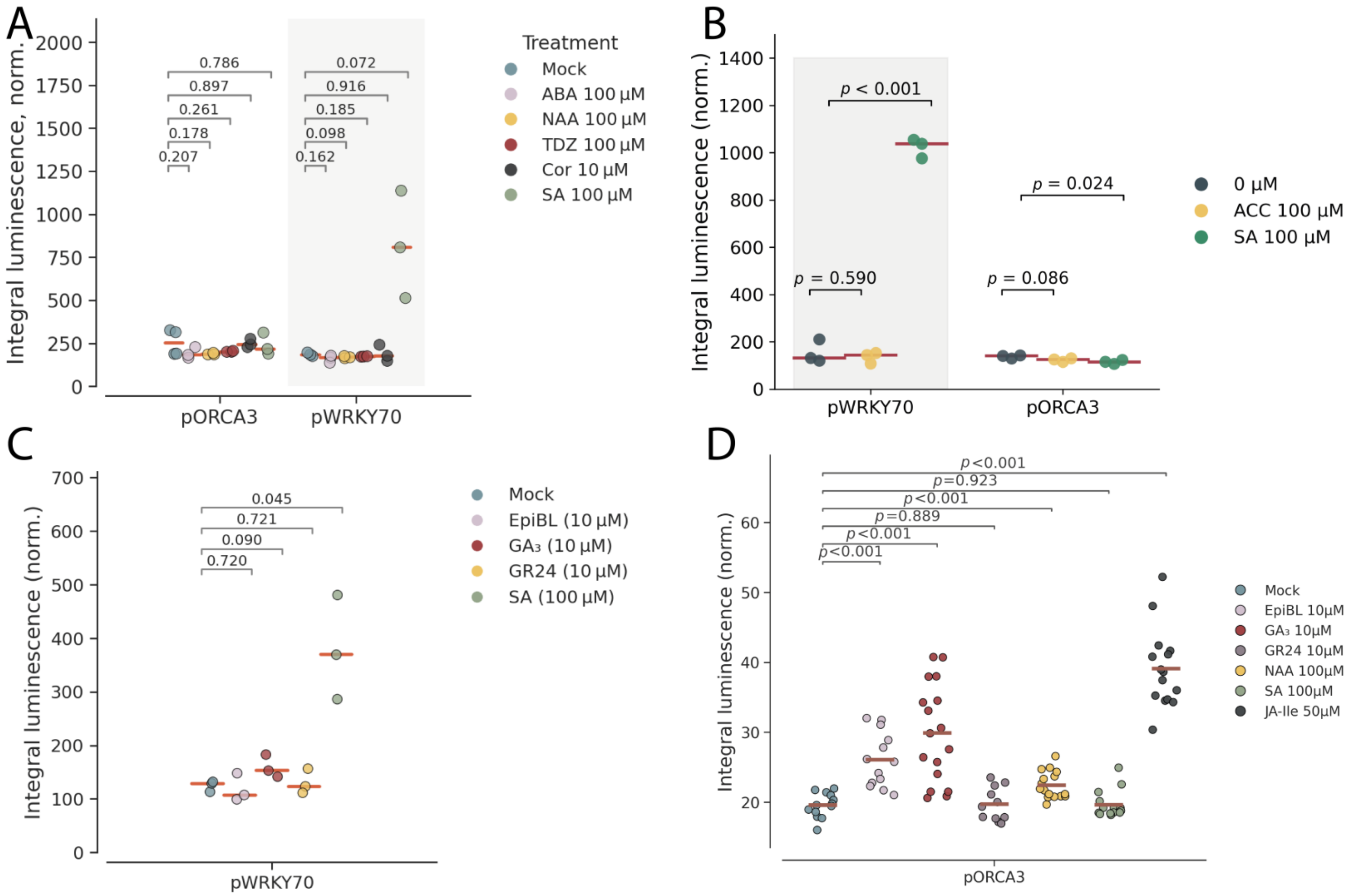

Supplementary Figure 8. Assessment of luminescence levels in response to a range of treatments in stable lines of *N. benthamiana*. A. The result of treatment of *pORCA3-nnLuz* and *pWRKY70-nnLuz* lines with ABA (abscisic acid, 100 µM), NAA (1-naphthaleneacetic acid, 100µM), thidiazuron (TDZ, 100µM), coronatine (Cor, 10µM), salicylic acid (SA, 100µM). B. The result of treatment of *pORCA3-nnLuz* and *pWRKY70-nnLuz* lines with 1-aminocyclopropane-1-carboxylic acid (ACC, 100µM) or salicylic acid (SA, 100µM). C. The result of treatment of *pWRKY70-nnLuz* lines with epibrassinolide (EpiBL, 10µM), gibberellin GA3 (GA3, 10µM), strigolactone GR24 (GR24, 20µM), or salicylic acid (SA, 100µM). D. The result of treatment of *pORCA3-nnLuz* with epibrassinolide (EpiBL, 10µM), gibberellin GA3 (GA3, 10µM), strigolactone GR24 (GR24, 20µM), NAA (1-naphthaleneacetic acid, 100µM), salicylic acid (SA, 100µM), or jasmonate isoleucine (JA-Ile, 50µM). *p*-values were calculated with Welch's two-sample *t*-test, comparing the integral luminescence of the control (Mock) against each other treatment within the same promoter. Leaves of 4-week-old plants (A, C, D) or 1-week-old plants (B) were used in these experiments. Compound solutions were applied to the leaves or plants once at the start of the experiment, the imaging lasted for 24 hours (A, C, D) or 48 hours (B). Source data are provided as a Source Data file.

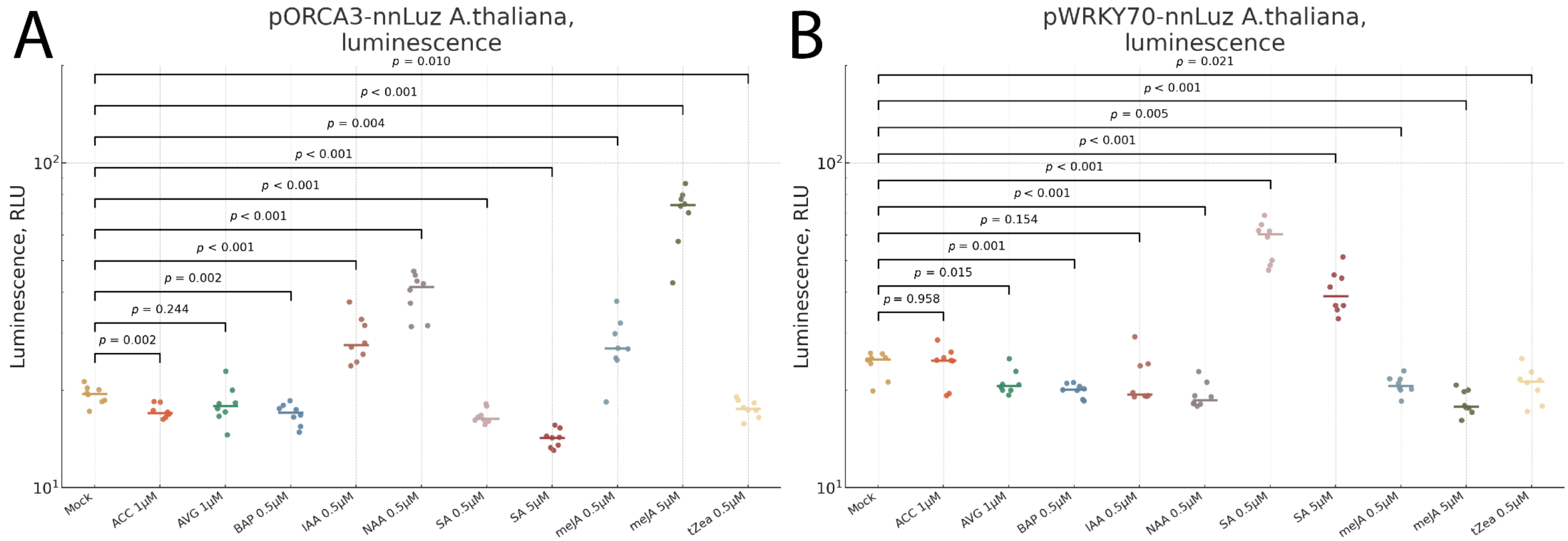

Supplementary Figure 9. Assessment of luminescence levels in response to a range of treatments in stable lines of *A. thaliana.* The result of treatment of *A. thaliana* plants – A. pORCA3-nnLuz-expressing and B. pWRKY70-nnLuz-expressing plants – with a range of phytohormones, analogs or other potential inducers. N = 8 biological replicates. *p*-values were calculated with Welch's two-sample *t*-test, comparing the integral luminescence of the control (Mock) against each other treatment within the same promoter. The experiments were conducted on 6-day-old plants. Mock — untreated control, ACC – 1-aminocyclopropane-1-carboxylic acid, IAA— indole-3-acetic acid, NAA — 1-naphthaleneacetic acid, BAP — 6-benzylaminopurine, tZea — trans-zeatin, AVG — aminoethoxyvinylglycine, meJa — methyl jasmonate, SA — salicylic acid. Source data is provided as a Source Data file.

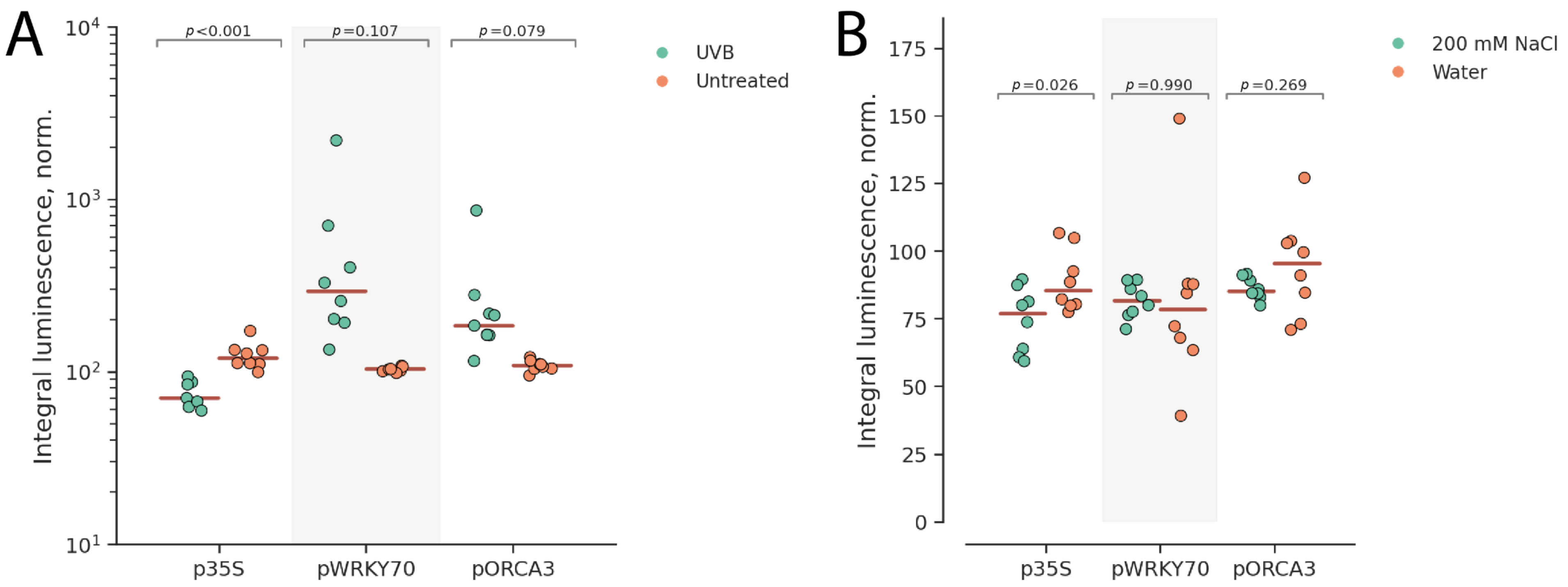

Supplementary Figure 10. The result of treatment of *N. benthamiana* salicylic acid (*pWKRY70-nnLuz-WRKY70_T*) or jasmonic acid (*pORCA3-nnLuz-ORCA3_T*) reporters with A. UVB or B. salt. NaCl treatment was performed by watering the plants for 20 seconds with 200 mM solution once until the water came out of the pot and immediately started imaging. For UVB treatment the plants were exposed to 312 nm UV light (Vilber Lourmat) for 30 minutes. N = 8 technical replicates. *p*-values were calculated with Welch's two-sample *t*-test, comparing the integral luminescence of the control (Untreated or Water) against the other treatment within the same promoter. The experiments were conducted on 4-week-old plants. Source data are provided as a Source Data file.

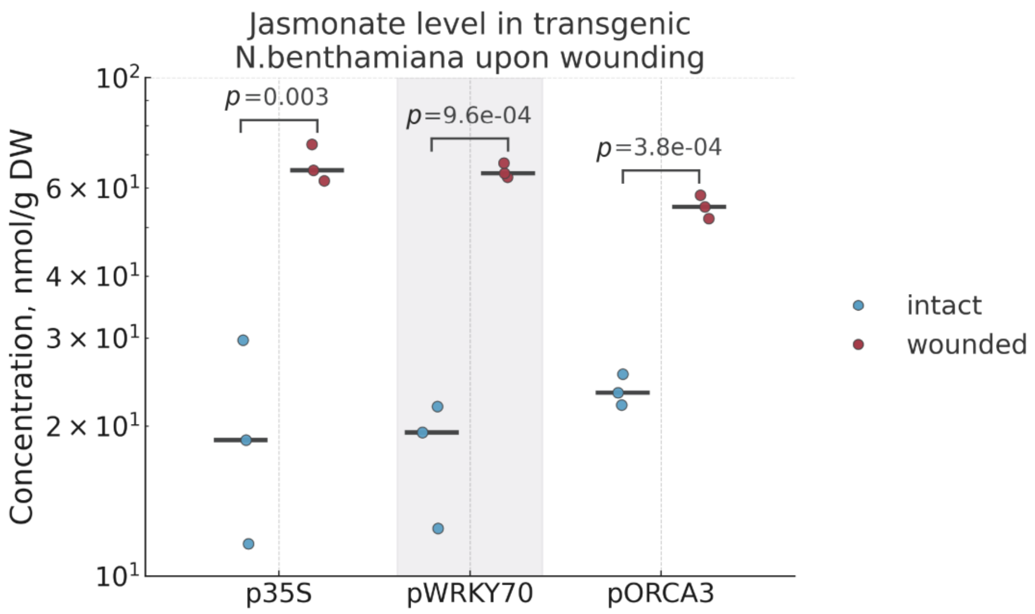

Supplementary Figure 11. Jasmonic acid levels in *N. benthamiana* salicylic acid (*pWKRY70-nnLuz-WRKY70_T*) or jasmonic acid (*pORCA3-nnLuz-ORCA3_T*) reporter plants 5 hours after leaf wounding with scissors for 10 seconds, as identified by LC-MS. Swarms correspond to biological replicates. The black line is the median, the coloured points represent individual data points. N = 3 independent biological samples. *p*-values were calculated with Welch's two-sample *t*-test. Source data are provided as a Source Data file.

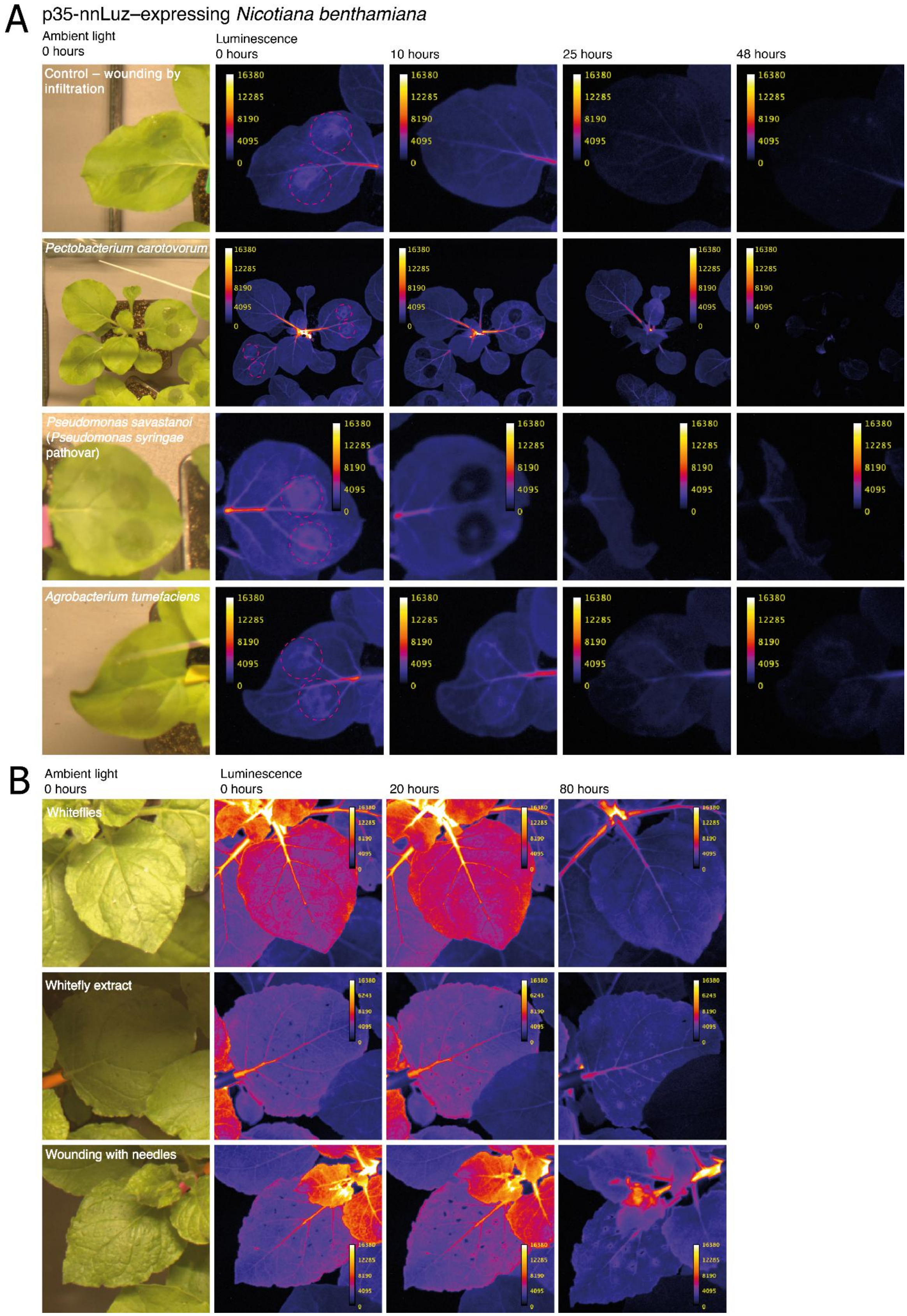

Supplementary Figure 12. Luminescence of control *p35S-nnLuz*-expressing 4-week-old *Nicotiana benthamiana* upon exposure to pests and pathogens. Luminescence from plants infiltrated with A.) buffer, hemibiotroph *Pseudomonas savastanoi*, necrotrophic bacteria *Pectobacterium carotovorum,* or *Agrobacterium tumefaciens.* Infiltrations were done once at the start of the experiment and are indicated as circles with a pink dashed line. B.) Visible light and luminescence images at different time points upon exposure to whitefly bites, and to wounding with needles dipped in whitefly extract, or in buffer. The experiment was repeated 2 times with the same result.

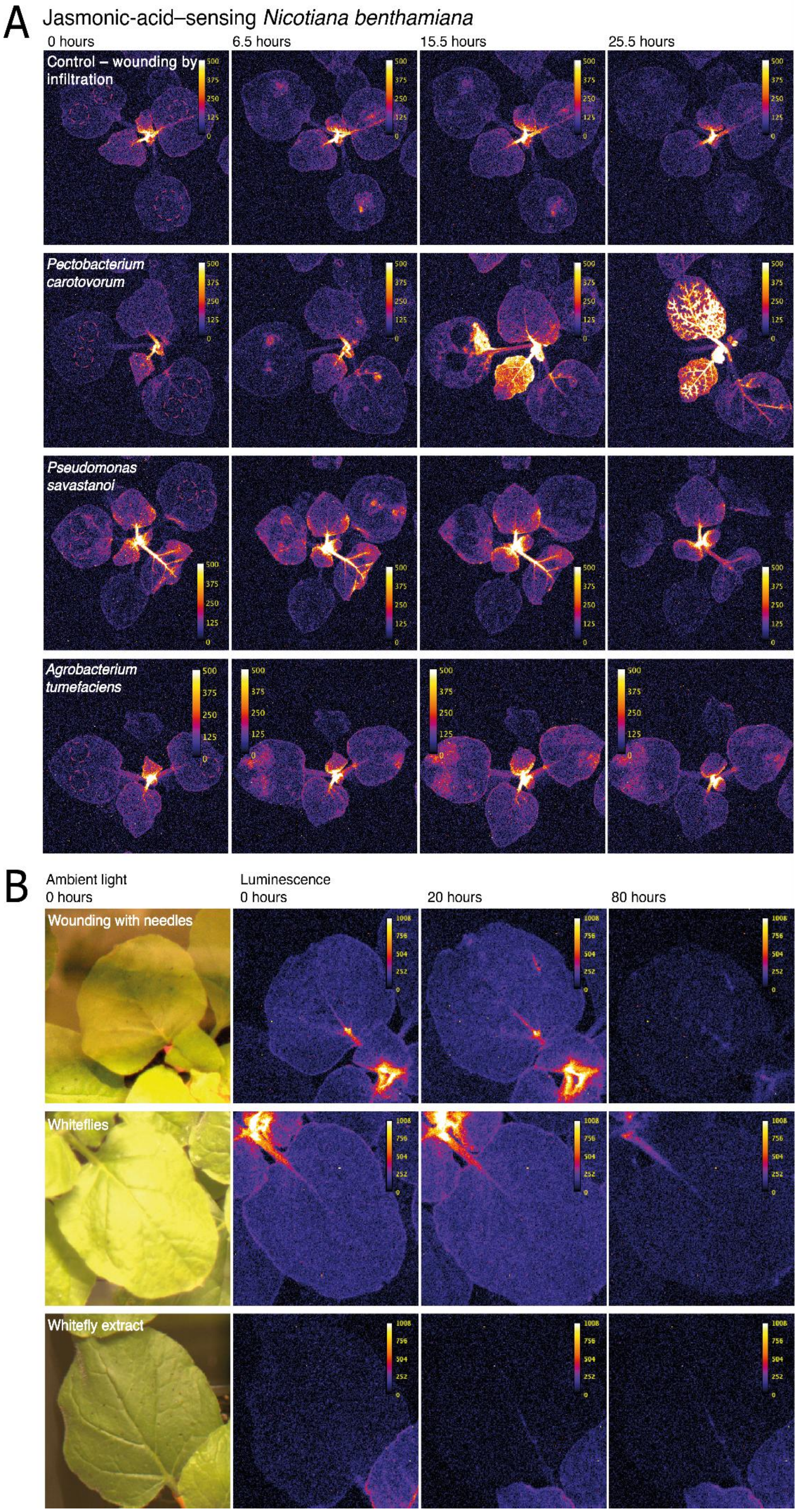

Supplementary Figure 13. The response of *pORCA3-nnLuz-ORCA3_T*-expressing transgenic 4-week-old *Nicotiana benthamiana* plants. Luminescent images corresponding to different time points for the development of luminescent signals in response to A. infiltration with buffer, hemibiotroph *Pseudomonas savastanoi*, necrotrophic bacteria *Pectobacterium carotovorum,* or *Agrobacterium tumefaciens,* and are indicated as circles with a pink dashed line; B. whitefly bites, whitefly extract and wounding, or and wounding only. Treatments were done once at the start of the experiment. The experiment was repeated 2 times with the same result.

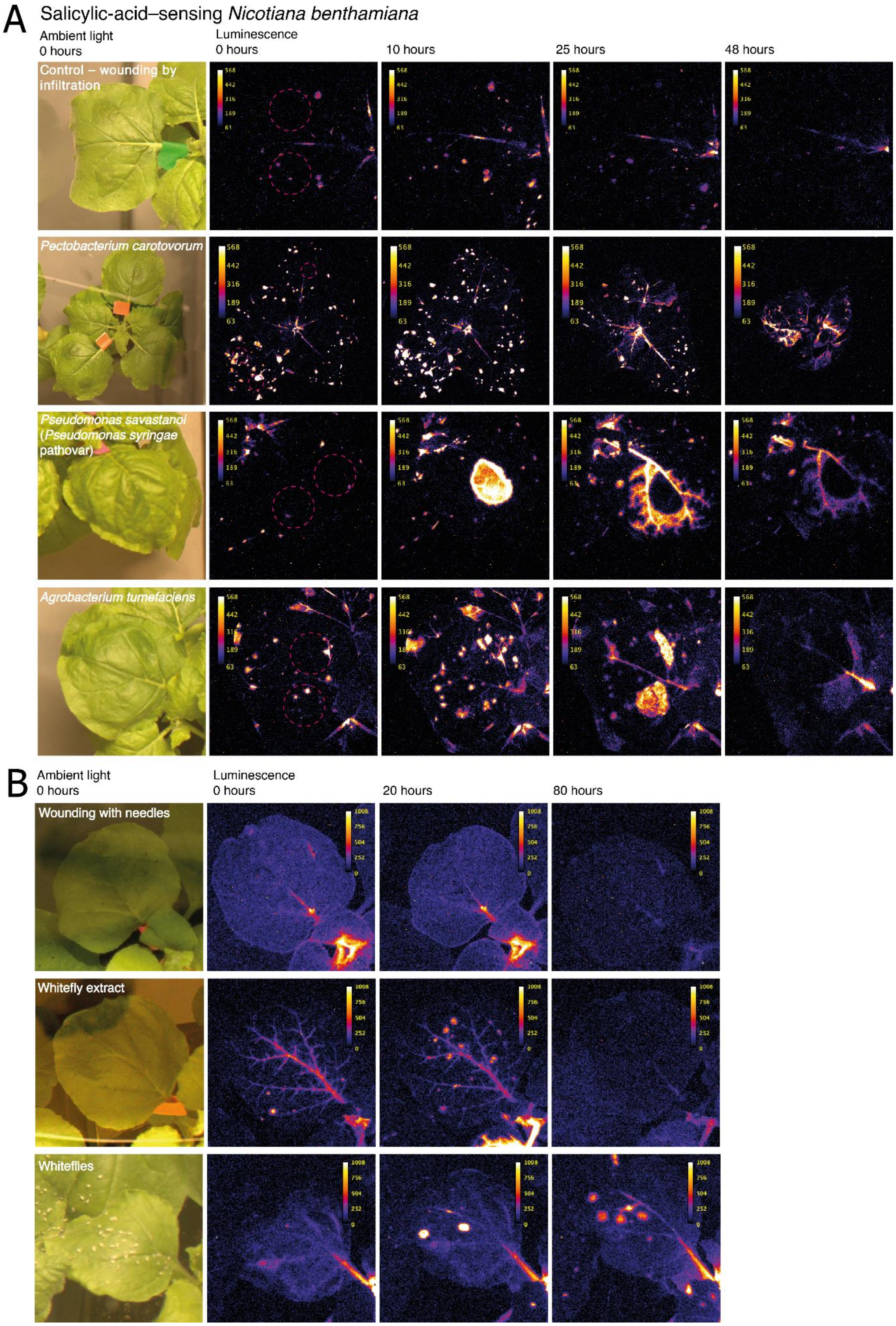

Supplementary Figure 14. The response of *pWRKY70-nnLuz-WRKY70_T*-expressing 4-week-old transgenic *Nicotiana benthamiana* plants. Visible light image and luminescent images corresponding to different time points for the development of luminescent signals in response to A. infiltration with buffer, hemibiotroph *Pseudomonas savastanoi*, necrotrophic bacteria *Pectobacterium carotovorum,* or *Agrobacterium tumefaciens,* and are indicated as circles with a pink dashed line; B. whitefly bites, whitefly extract and wounding, or wounding only. Treatments were done once at the start of the experiment. The experiment was repeated 2 times with the same result.

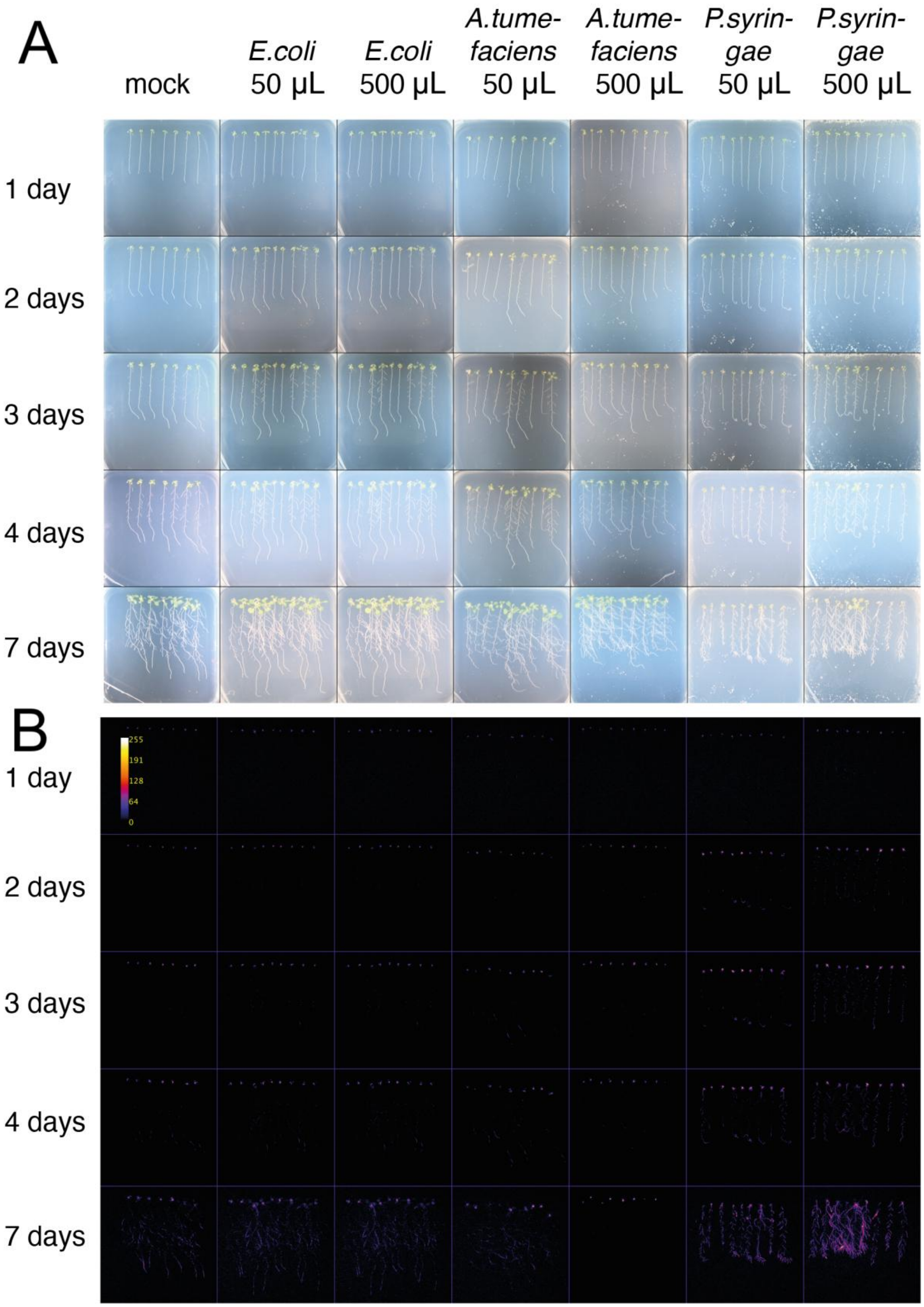

Supplementary Figure 15. The complete imaging of the *in vitro* infection assay of stably transformed *pORCA3* bioluminescent *A. thaliana* reporters with *E. coli*, *A. tumefaciens*, *P. syringae* DC3000 in a 7-day experiment. The images taken in ambient light (A) and in the dark (B). N= 9 biological samples. The bottommost row corresponds to Figure 2a in the main text.

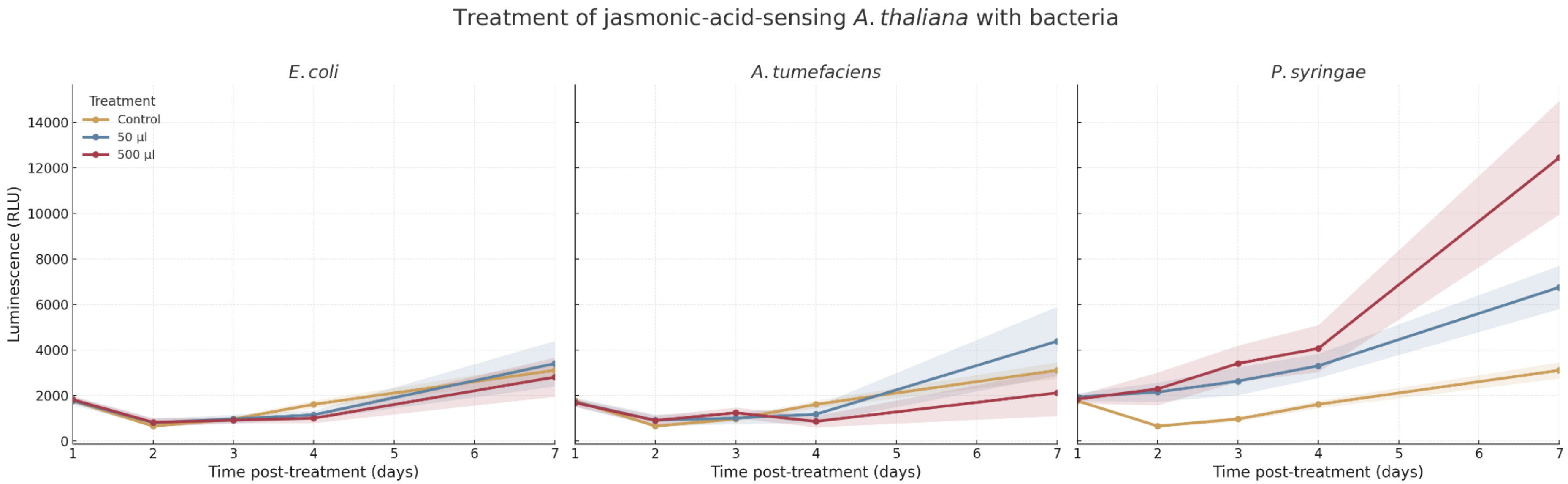

Supplementary Figure 16. Quantification of the complete imaging of the *in vitro* infection assay of stably transformed *pORCA3* bioluminescent *A. thaliana* reporters with *E.coli*, *A. tumefaciens*, *P. syringae* DC3000 in a 7-day experiment. N = 9 biological replicates for each treatment. Error bars represent mean values +/- SD. Source data are provided as a Source Data file.

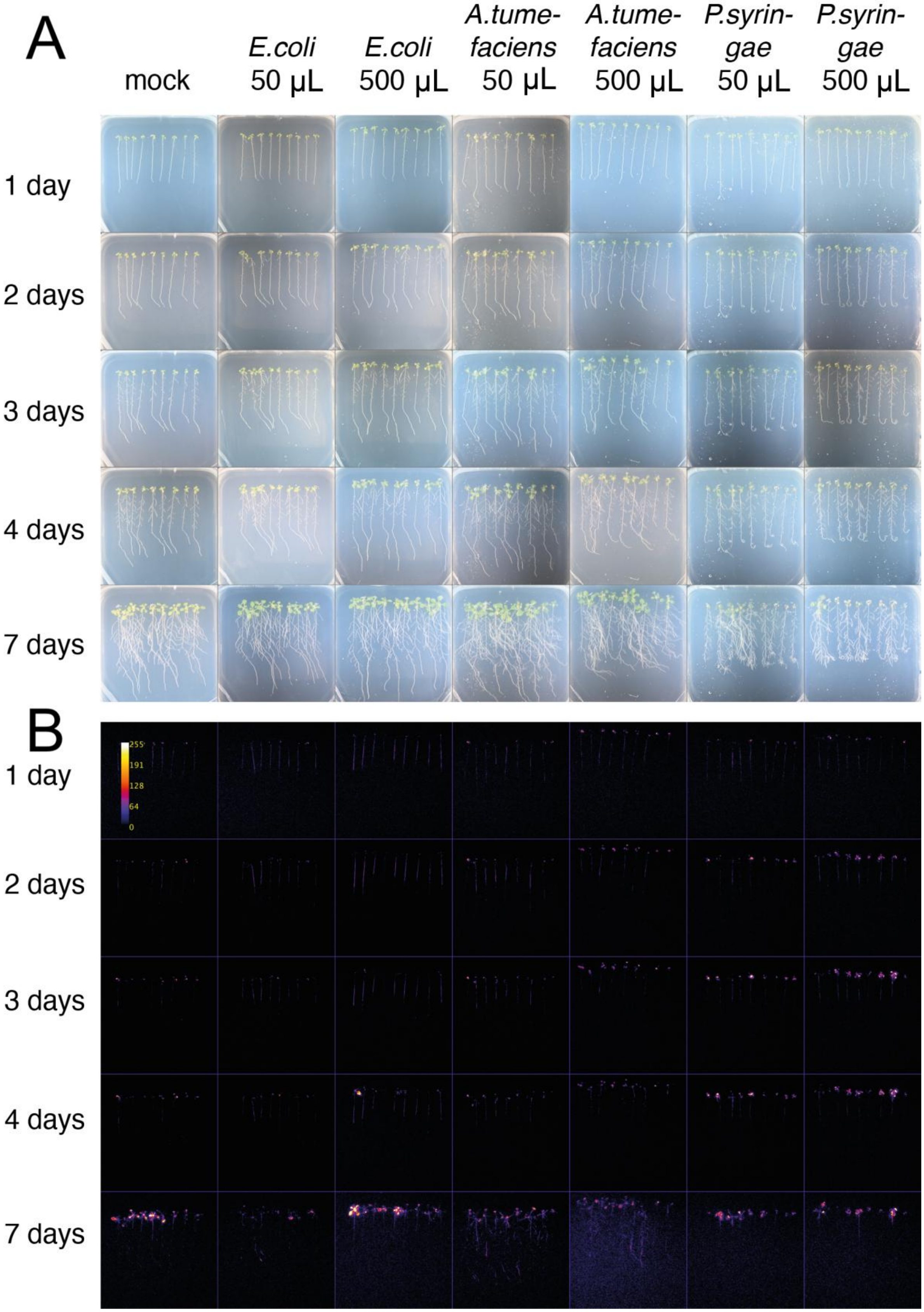

Supplementary Figure 17. The complete imaging of the *in vitro* infection assay of stably transformed *pWRKY70* bioluminescent *A. thaliana* reporters with *E.coli*, *A. tumefaciens*, *P. syringae* DC3000 in a 7-day-experiment. The images taken in ambient light (A) and in the dark (B). N= 9 biological samples.

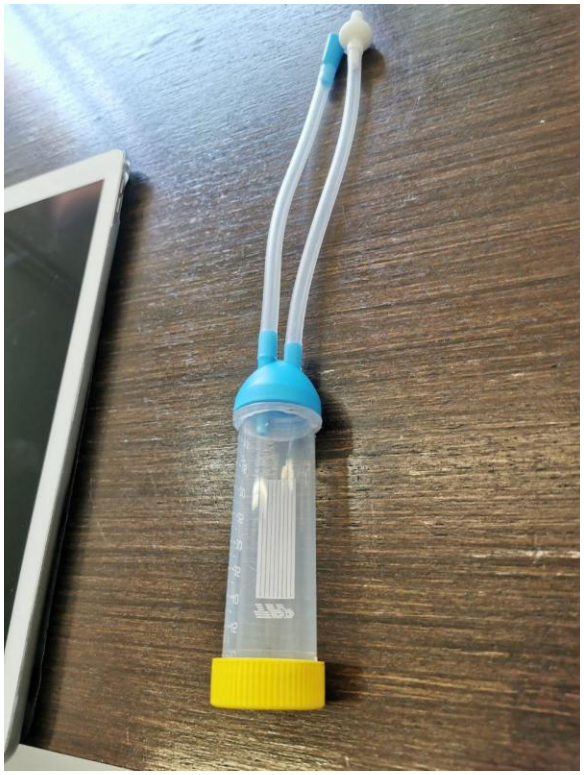

Supplementary Figure 18. A custom aspirator used to capture whiteflies.

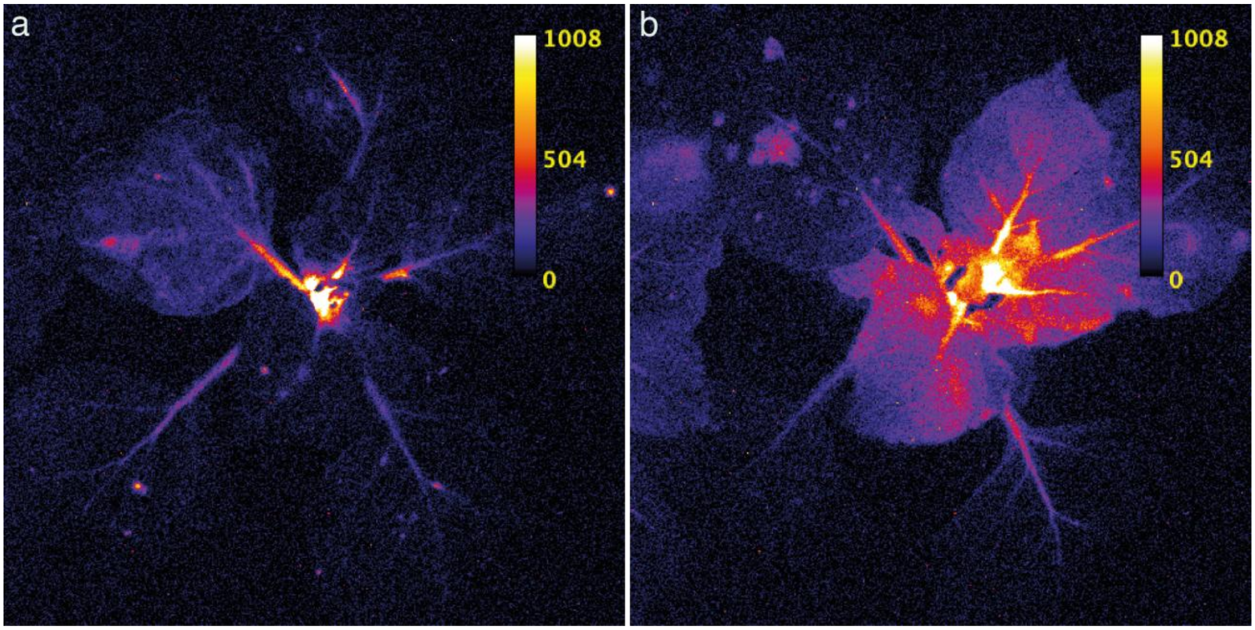

Supplementary Figure 19. Luminescence of *pWRKY70-nnLuz-WRKY70_T* salicylic-acid-sensing 4-week-old *N. benthamiana* plants at the start (a) and at the end (7 day, b) of the whitefly exposure experiment. N = 4 biological replicates.

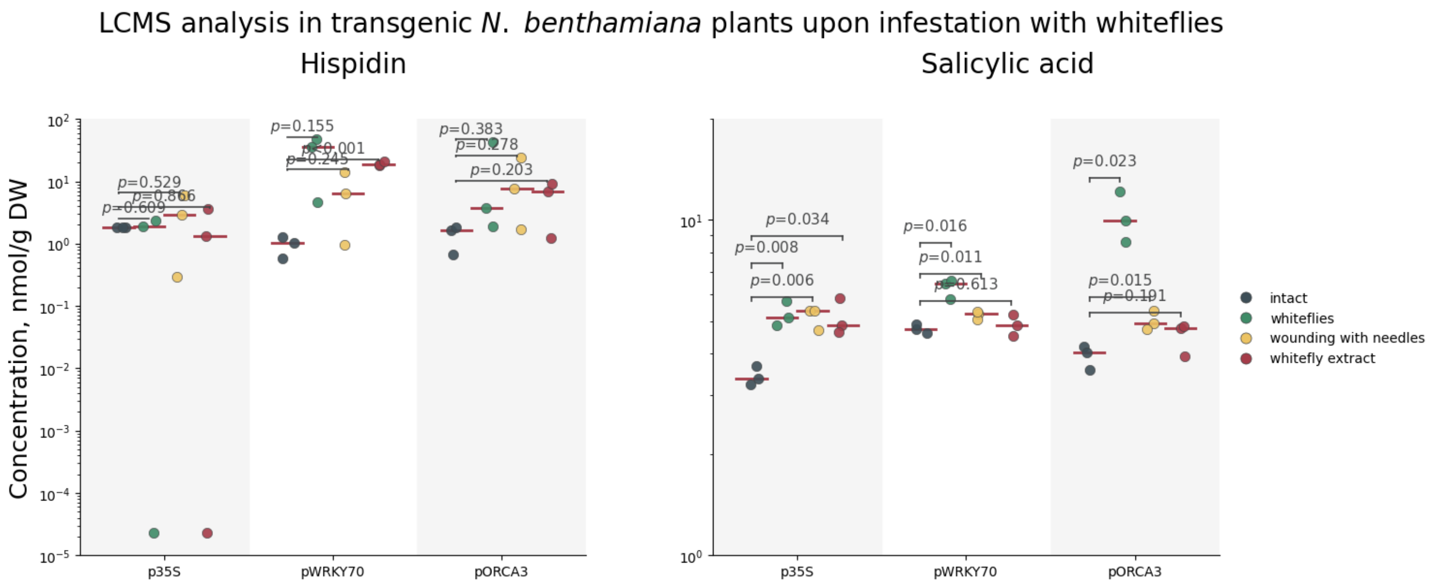

Supplementary Figure 20. The levels of a major fungal bioluminescence metabolite hispidin and salicylic acid upon infestation with whiteflies with LC-MS. Reporters utilised – pWRKY70-nnLuz-WRKY70_T, pORCA3-nnLuz-ORCA3_T, control p35S-nnLuz-Act2. The red line is the median, the coloured points represent individual data points. N = 3 biologically independent samples. *p*-values were calculated with Welch's two-sample *t*-test. Source data are provided as a Source Data file.

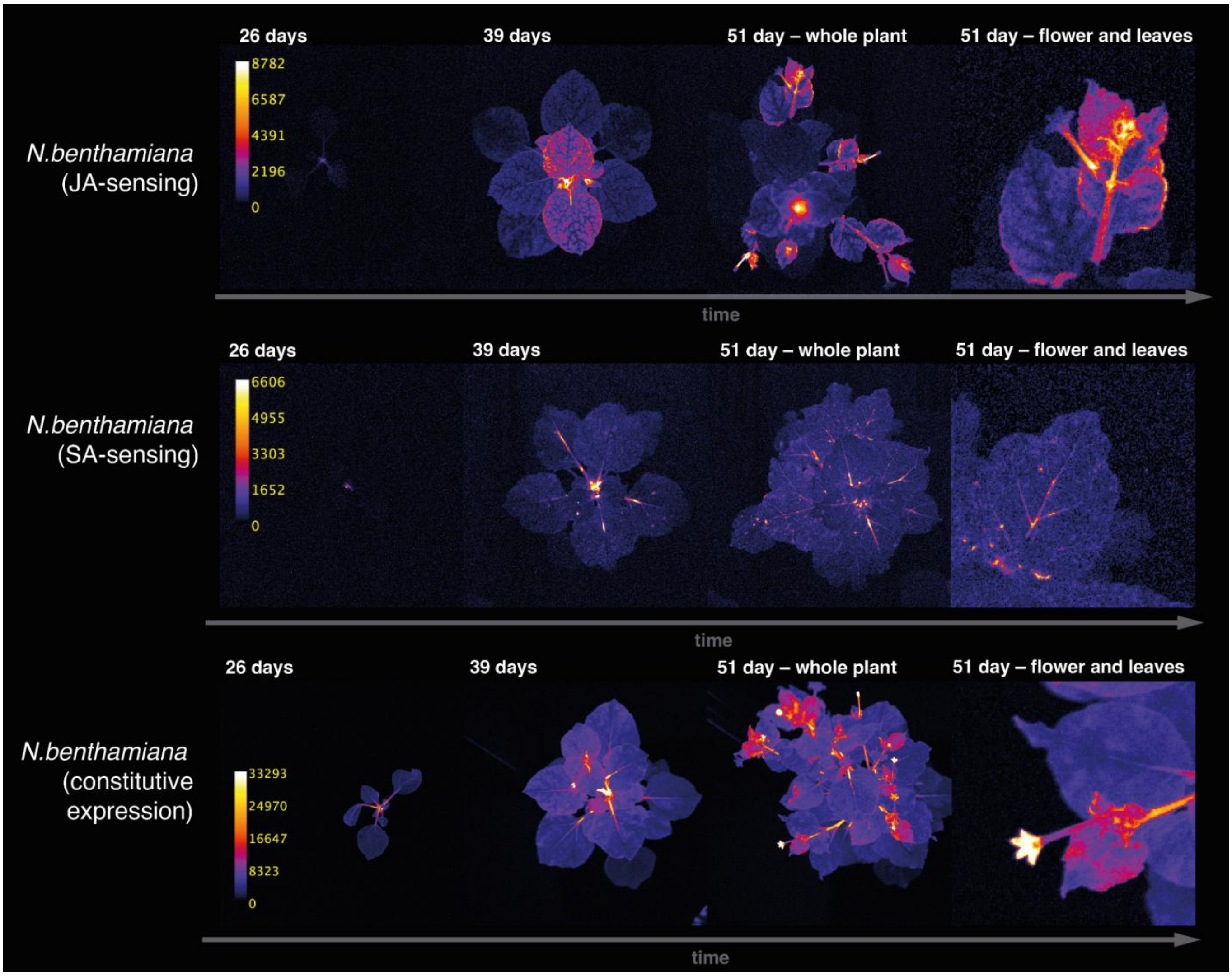

Supplementary Figure 21. Performance of reporters of salicylic, jasmonic acids or constitutively expressing luminescence genes in transgenic *Nicotiana benthamiana* plants during normal growth and flowering. Reporters utilised – pWRKY70-nnLuz-WRKY70_T, pORCA3-nnLuz-ORCA3_T, control p35S-nnLuz-Act2. N = 2 biological replicates, experiment was repeated two times.

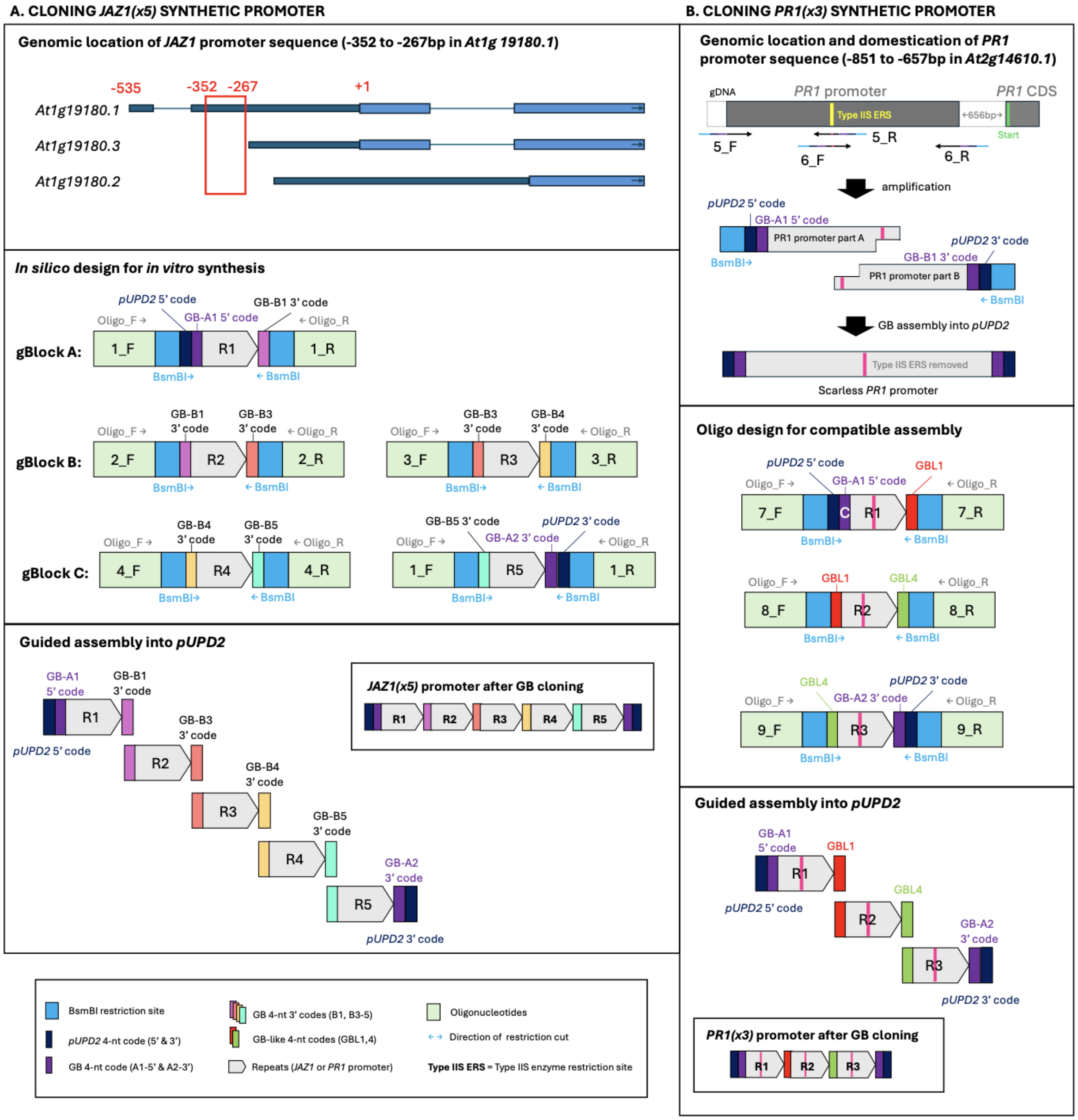

Supplementary Figure 22. Synthetic repetitive promoter assembly pipeline for *JAZ1(x5)* and *PR1(x3)*. A. Cloning strategy for the JAZ1(x5) synthetic promoter. The desired *JAZ1* sequence (red rectangle) is found at nucleotides -352 to -267 upstream of ATG in the 5'UTR of *JAZ1* cDNA isoform 1 and in the promoter of *JAZ1* cDNA isoforms 2 and 3. As JAZ1(x5) commercial synthesis failed due to the sequence of interest not passing sequence complexity filters, an alternative pipeline was developed. Individual repeats were synthesized commercially with complementary 4 nt codes from the GoldenBraid (GB) grammar [1](in pink B1 3' code [CCAT], salmon B3 3'code [AGCC], yellow B4 3'code [TTCG], and cyan B5 3' code [GCTT]), BsmBI restriction sites (blue), and unique oligo pairs (light green) at their flanks. These sequences were ordered as part of bigger IDT gBlocks containing additional unrelated sequences for other projects. The complementarity between the 4nt codes allowed a guided and directional assembly of the five repeats simultaneously in the GB pUPD2 entry clone, producing a clone with the final JAZ1(x5) synthetic promoter. B. Cloning strategy for the PR1(x3) synthetic promoter. Initial domestication for the 195 bp-long promoter sequence, found at nucleotides -851 to -657 upstream of ATG of the *PR1* gene, was needed to eliminate a GB type IIS enzyme restriction site (ERS, yellow line). For that purpose, two sets of oligo pairs were designed to amplify half of the sequence each and introducing a point mutation that disrupts the restriction site (pink line). Both amplified fragments were co-assembled into the GB pUPD2 entry clone in a scarless manner and then used as a template for creating the promoter repeats. Each repeat was flanked by similar elements as for the *JAZ1* repeats but using two GB-like 4 nt codes (GBL1 [CAGT] in red, and GBL4 [AGCA] in green, previously described in [2]) instead of the regular GB 4nt codes. The complementarity between the GBL codes allowed a guided and directional subcloning of the three repeats simultaneously into pUPD2 creating the final clone harboring the full PR1(x3) synthetic promoter. Further details on cloning overhang information and multi-assembly procedures can be found in [2]. Oligo sequences can be found in Supplementary Table 3. CDS, protein coding sequence.

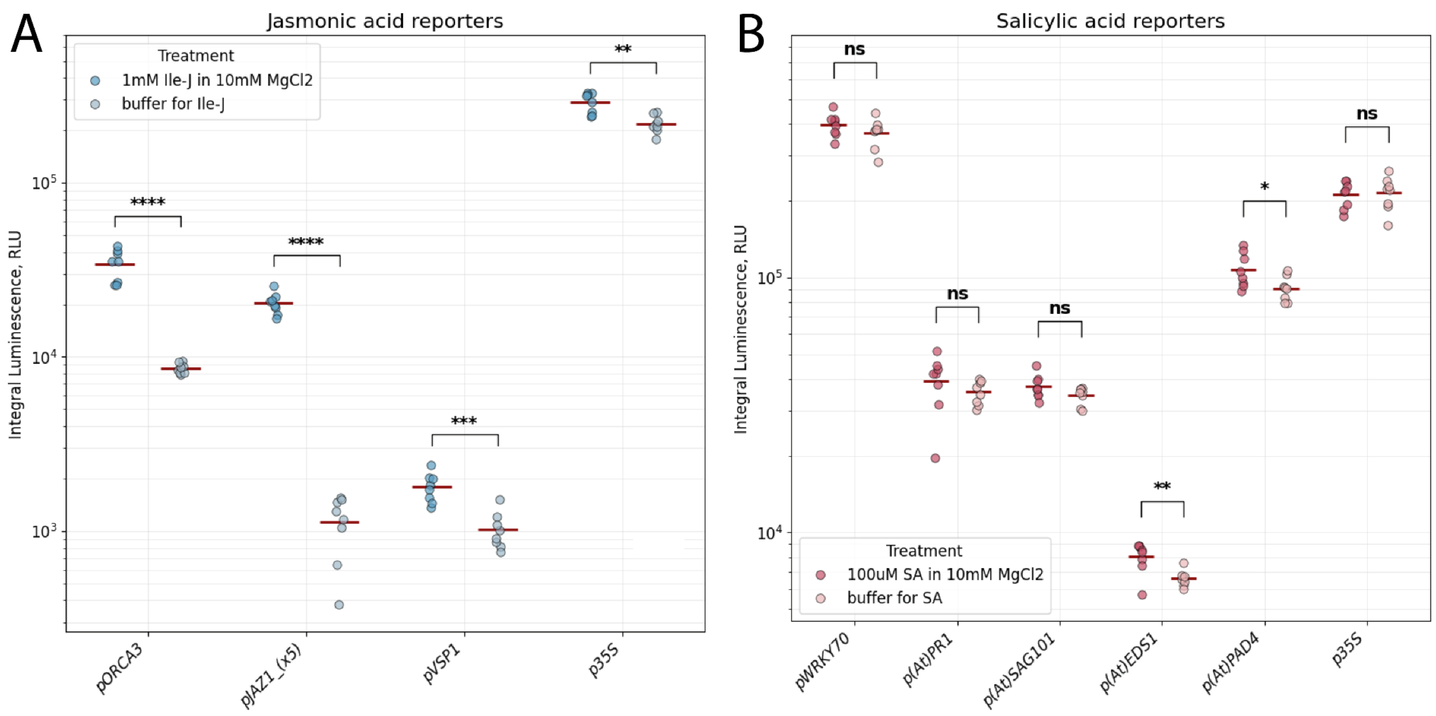

Supplementary Figure 23. The result of transient expression of transcriptional reporters of jasmonic acid (A) and salicylic acid (B) in BY-2 cells. The experiments were conducted on BY-2 cell packs. Jasmonic acid reporters were induced with 1 mM jasmonate isoleucine for 30 minutes before imaging. Based on previous experiments, SA signaling could not be assayed in our transient overexpression assay, as agrobacterial infiltration itself induced SA signaling. Nevertheless, we treated the cell packs with 100μM salicylic acid solution. N = 8 technical replicates. We used a one-sided Mann-Whitney test to compare the integral luminescence values between the specific treatment and the corresponding control. To assess the difference between all the treatments within one genotype, an ANOVA was employed, followed by a Tukey HSD. Source data are provided as a Source Data file.

Supplementary Table 1. Plant lines used in the study.

Line ID	Line name	Inserts
NB237	*N. benthamiana* luciferase-less masterline	pNos-KanR-ocsT | p35S-nnHispS-ocsT | pCmYLCV-npgA-ATPT | p35S-nnCPH-ocsT | pFMV-nnH3H-nosT
NB4717	*N. benthamiana* salicylic-acid-sensing line	pNos-KanR-ocsT | p35S-nnHispS-ocsT | pCmYLCV-npgA-ATPT | p35S-nnCPH-ocsT | pFMV-nnH3H-nosT<br><br>pNOS-HygR-ocsT | pWRKY70-nnLuz-WRKY70_T
NB4776	*N. benthamiana* jasmonic-acid-sensing line	pNos-KanR-ocsT | p35S-nnHispS-ocsT | pCmYLCV-npgA-ATPT | p35S-nnCPH-ocsT | pFMV-nnH3H-nosT<br><br>pNOS-HygR-ocsT | pORCA3-nnLuz-ORCA3_T
NB4768	*N. benthamiana* autoluminescent line	pNos-KanR-ocsT | p35S-nnHispS-ocsT | pCmYLCV-npgA-ATPT | p35S-nnCPH-ocsT | pFMV-nnH3H-nosT<br><br>pNOS-HygR-ocsT | p35S-nnLuz-act2T
AT8462	*A. thaliana* luciferase-less masterline	pNos-KanR-ocsT | p35S-nnHispS-ocsT | pCmYLCV-npgA-ATPT | p35S-nnCPH-ocsT | pFMV-nnH3H-nosT
AT8463	*A. thaliana* salicylic-acid-sensing line	pNos-KanR-ocsT | p35S-nnHispS-ocsT | pCmYLCV-npgA-ATPT | p35S-nnCPH-ocsT | pFMV-nnH3H-nosT<br><br>pNOS-HygR-ocsT | pWRKY70-nnLuz-WRKY70_T
AT8464	*A. thaliana* jasmonic-acid-sensing line	pNos-KanR-ocsT | p35S-nnHispS-ocsT | pCmYLCV-npgA-ATPT | p35S-nnCPH-ocsT | pFMV-nnH3H-nosT<br><br>pNOS-HygR-ocsT | pORCA3-nnLuz-ORCA3_T

Supplementary Table 2. Primers used for qPCR of genes in *N. benthamiana*.

Gene	Forward primer	Reverse primer
nnLuz	tagtctcttcggataattgggaatgctattgc	tgacggtctcCaatgAGGtctactATCTCTTTGTCTAGTCT
EF1a	CTGCAACAAGATGGATGCTAC	CAAAACCAGAGATGGGGACGAA
PR-1a	CCTCGTACATTCTCATGGTCAAT	CCATTGTTACACTGAACCCTAGC
NbJAZ3	GTCAAGCAAGGTGGGTAT	GCTAATGTTAAACGGGAG

Supplementary Table 3. Oligonucleotides used to amplify the JAZ1 and PR1 elements to build JAZ1(x5) and PR1(x3) synthetic distal promoters.

**Oligos to amplify individual *JAZ1* repeats**	
primer 1_F	AATTAACCCTCACTAAAGGG
primer 1_R	GCCCTATAGTGAGTCGTATTAC
primer 2_F	CGAGATTGATCCAGTCGCAG
primer 2_R	GTCCGATTGCTAGATGTCGTTG
primer 3_F	GCAATGCAATAGGATCTCTACGAC
primer 3_R	CTCCATCAATCTTCGCCTGCAAC
primer 4_F	CCAAAGCCATCACCCTCGAC
primer 4_R	GACATGACCTTATTTTTGACGCTACGCTGAC
**Oligos to domesticate *PR1* promoter**	
primer 5_F	GCGCCGTCTCGCTCGGGAGGTCACCTAGAGTTTTTCAATTTAAAC
primer 5_R	GCGCCGTCTCGTTTTCTATTTCAAATTTGAATTTCATATATTAC
primer 6_F	GCGCCGTCTCGAAAACTTAAATTAGAATCATGAAGAAAAAAAA
primer 6_R	GCGCCGTCTCGCTCAATGGATTTTGGGGTTCGTAAACGTCG
**Oligos to amplify individual domesticated *PR1* repeats**	
primer 7_F	GCGCCGTCTCACTCGGGAGGTCACCTAGAGTTTTTCAATTTAAAC
primer 7_R	GCGCCGTCTCGACTGATATATGCCGCCACATCTATG
primer 8_F	GCGCCGTCTCGCAGTGTCACCTAGAGTTTTTCAATTTAAAC
primer 8_R	GCGCCGTCTCGTGCTATATATGCCGCCACATCTATG
primer 9_F	GCGCCGTCTCGAGCAGTCACCTAGAGTTTTTCAATTTAAAC
primer 9_R	GCGCCGTCTCACTCAGGGAATATATGCCGCCACATCTATG

Supplementary references

1. Sarrion-Perdigones, A. *et al.* GoldenBraid 2.0: a comprehensive DNA assembly framework for plant synthetic biology. *Plant Physiol*. **162**, 1618–1631 (2013).
2. Fernandez-Moreno, J.-P. *et al.* A rapid and scalable approach to build synthetic repetitive hormone-responsive promoters. *Plant Biotechnol. J*. **22**, 1942–1956 (2024).
