# Supplementary material for: Non-invasive imaging of defence responses in plants
Source: Nat Commun. 2026 Mar 13;17:6393. doi: 10.1038/s41467-026-70075-1 (PMC13376918; doi:10.1038/s41467-026-70075-1)
Supplement: Supplementary file 3 — Description of Additional Supplementary Files [file 41467_2026_70075_MOESM3_ESM.pdf]

Description of Additional Supplementary Files

File Name: Supplementary Data 1
Description: Plasmids used in the study.

File Name: Supplementary Data 2
Description: Additional stimuli affecting pORCA3 and pWRKY70 promoters

File Name: Supplementary Movie 1
Description: A 2-day-long timelapse of a *pORCA3-nnLuz*-expressing 4-week-old *Nicotiana benthamiana* leaf upon wounding – infiltration with buffer (upper left), infiltration of *Pseudomonas savastanoi* (upper right), infiltration of *Pectobacterium carotovorum* (lower left), and infiltration of *Agrobacterium tumefaciens* (lower right). Pictures taken with Sony Alpha ILCE-7M3 camera and 35-mm T1.5 ED AS UMC VDSLR lens (Samyang, ~f/1.4) with an exposure of 30 s and ISO 3,200. Imaging performed every 30 minutes.

File Name: Supplementary Movie 2
Description: A 2-day-long timelapse of a *p35S-nnLuz*-expressing 4-week-old *Nicotiana benthamiana* leaf upon wounding – infiltration with buffer (upper left), infiltration of *Pseudomonas savastanoi* (upper right), infiltration of *Pectobacterium carotovorum* (lower left), and infiltration of *Agrobacterium tumefaciens* (lower right). Pictures taken with Sony Alpha ILCE-7M3 camera and 35-mm T1.5 ED AS UMC VDSLR lens (Samyang, ~f/1.4) with an exposure of 30 s and ISO 3,200. Imaging performed every 30 minutes.

File Name: Supplementary Movie 3
Description: A 2-day-long timelapse of a *pWRKY70-nnLuz*-expressing 4-week-old *Nicotiana benthamiana* leaf upon wounding – infiltration with buffer (upper left), infiltration of *Pseudomonas savastanoi* (upper right), infiltration of *Pectobacterium carotovorum* (lower left), and infiltration of *Agrobacterium tumefaciens* (lower right). Pictures taken with Sony Alpha ILCE-7M3 camera and 35-mm T1.5 ED AS UMC VDSLR lens (Samyang, ~f/1.4) with an exposure of 30 s and ISO 3,200. Imaging performed every 30 minutes.

File Name: Supplementary Movie 4
Description: A 7-day-long timelapse of a *p35S-nnLuz*-expressing 4-week-old *Nicotiana benthamiana* leaf after wounding with needles. Pictures taken with Sony Alpha ILCE-7M3 camera and 35-mm T1.5 ED AS UMC VDSLR lens (Samyang, ~f/1.4) with an exposure of 30 s and ISO 3,200. Imaging performed every 30 minutes.

File Name: Supplementary Movie 5
Description: A 7-day-long timelapse of a *p35S-nnLuz*-expressing 4-week-old *Nicotiana benthamiana* leaf after wounding with needles dipped in whitefly extract. Pictures taken with Sony Alpha ILCE-7M3 camera and 35-mm T1.5 ED AS UMC VDSLR lens (Samyang, ~f/1.4) with an exposure of 30 s and ISO 3,200. Imaging performed every 30 minutes.

File Name: Supplementary Movie 6
Description: A 7-day-long timelapse of a *p35S-nnLuz*-expressing 4-week-old *Nicotiana benthamiana* leaf after whitefly infestation. Pictures taken with Sony Alpha ILCE-7M3 camera and 35-mm T1.5 ED AS UMC VDSLR lens (Samyang, ~f/1.4) with an exposure of 30 s and ISO 3,200. Imaging performed every 30 minutes.

File Name: Supplementary Movie 7
Description: A 7-day-long timelapse of a *pORCA3-nnLuz*-expressing 4-week-old *Nicotiana benthamiana* leaf after wounding with needles. Pictures taken with Sony Alpha ILCE-7M3 camera and 35-mm T1.5 ED AS UMC VDSLR lens (Samyang, ~f/1.4) with an exposure of 30 s and ISO 3,200. Imaging performed every 30 minutes.

File Name: Supplementary Movie 8
Description: A 7-day-long timelapse of a *pORCA3-nnLuz*-expressing 4-week-old *Nicotiana benthamiana* leaf after wounding with needles dipped in whitefly extract. Pictures taken with Sony Alpha ILCE-7M3 camera and 35-mm T1.5 ED AS UMC VDSLR lens (Samyang, ~f/1.4) with an exposure of 30 s and ISO 3,200. Imaging performed every 30 minutes.

File Name: Supplementary Movie 9
Description: A 7-day-long timelapse of a *pORCA3-nnLuz*-expressing 4-week-old *Nicotiana benthamiana* leaf after whitefly infestation. Pictures taken with Sony Alpha ILCE-7M3 camera and 35-mm T1.5 ED AS UMC VDSLR lens (Samyang, ~f/1.4) with an exposure of 30 s and ISO 3,200. Imaging performed every 30 minutes.

File Name: Supplementary Movie 10
Description: A 7-day-long timelapse of a *pWRKY70-nnLuz*-expressing 4-week-old *Nicotiana benthamiana* leaf after wounding with needles. Pictures taken with Sony Alpha ILCE-7M3 camera and 35-mm T1.5 ED AS UMC VDSLR lens (Samyang, ~f/1.4) with an exposure of 30 s and ISO 3,200. Imaging performed every 30 minutes.

File Name: Supplementary Movie 11
Description: A 7-day-long timelapse of a *pWRKY70-nnLuz*-expressing 4-week-old *Nicotiana benthamiana* leaf after wounding with needles dipped in whitefly extract. Pictures taken with Sony Alpha ILCE-7M3 camera and 35-mm T1.5 ED AS UMC VDSLR lens (Samyang, ~f/1.4) with an exposure of 30 s and ISO 3,200. Imaging performed every 30 minutes.

File Name: Supplementary Movie 12
Description: A 7-day-long timelapse of a *pWRKY70-nnLuz*-expressing 4-week-old *Nicotiana benthamiana* leaf after whitefly infestation. Pictures taken with Sony Alpha ILCE-7M3 camera and 35-mm T1.5 ED AS UMC VDSLR lens (Samyang, ~f/1.4) with an exposure of 30 s and ISO 3,200. Imaging performed every 30 minutes.

File Name: Supplementary Movie 13
Description: A 1.5-month-long timelapse of a *p35S-nnLuz*-expressing *Nicotiana benthamiana* plant. Pictures taken with Sony Alpha ILCE-7M3 camera and 35-mm T1.5 ED AS UMC VDSLR lens (Samyang, ~f/1.4) with an exposure of 30 s and ISO 400. Imaging performed every 30 minutes.

File Name: Supplementary Movie 14
Description: A 1.5-month-long timelapse of a *pORCA3-nnLuz*-expressing *Nicotiana benthamiana* plant. Pictures taken with Sony Alpha ILCE-7M3 camera and 35-mm T1.5 ED AS UMC VDSLR lens (Samyang, ~f/1.4) with an exposure of 30 s and ISO 400. Imaging performed every 30 minutes.

File Name: Supplementary Movie 15

Description: A 1.5-month timelapse of a *pWRKY70-nnLuz*-expressing *Nicotiana benthamiana* plant. Pictures taken with Sony Alpha ILCE-7M3 camera and 35-mm T1.5 ED AS UMC VDSLR lens (Samyang, ~f/1.4) with an exposure of 30 s and ISO 400. Imaging performed every 30 minutes.
