# Supplementary material for: Non-invasive imaging of defence responses in plants
Source: Nat Commun. 2026 Mar 13;17:6393. doi: 10.1038/s41467-026-70075-1 (PMC13376918; doi:10.1038/s41467-026-70075-1)
Supplement: Supplementary file 2 — Peer Review file [file 41467_2026_70075_MOESM2_ESM.pdf]

nature portfolio

Peer Review File

Non-invasive imaging of defence responses in plants

Corresponding Author: Dr Karen Sarkisyan

This file contains all reviewer reports in order by version, followed by all author rebuttals in order by version.

Version 0:

Reviewer comments:

Reviewer #1

(Remarks to the Author)
In this intriguing manuscript titled “Non-invasive Imaging of Salicylic and Jasmonic Acid Activities in Planta,” the authors developed a reporter system based on the FBP (fluorescent biosensor protein) platform. They reconstituted the overexpression of four fungal genes—HispS, NpgA, H3H, and CPH—and utilized the jasmonate-responsive pORCA3 promoter and the salicylic acid-responsive pWRKY70 promoter to drive the expression of Luz. This enabled the transgenic plants to monitor defense responses during pest and pathogen attacks. The proposed plant sensor demonstrates high sensitivity and efficacy, offering a novel conceptual framework for smart plant sensors. Overall, the manuscript presents compelling findings. However, certain issues need to be addressed to further substantiate the conclusions and enhance the robustness of the study.

1. Salicylic acid (SA) and jasmonic acid (JA) are pivotal plant hormones that regulate defense responses and developmental processes. Since the FBP module is integrated into the phenylpropanoid metabolic pathway, and both SA and JA are known to modulate this pathway, it is crucial to address how FBP-based reporters differentiate between phenylpropanoid metabolism (e.g., caffeic acid production) and biotic stress signaling (e.g., WRKY70 promoter-driven Luz expression) in response to pest and pathogen attacks. Elucidating the effects of SA and JA on the phenylpropanoid pathway could provide valuable insights into the utility of FBP-based reporters for monitoring plant defense under biotic stress conditions.
2. In Figure 2A and 2B, the current line colors in the line chart are challenging to distinguish. To improve clarity and visual accessibility, it is recommended to modify the color scheme to ensure better differentiation between the lines.
3. Based on the pest and pathogen signaling pathways, you selected the jasmonate-responsive promoter pORCA3 and the salicylic acid-responsive WRKY70 promoter to drive the expression of Luz. To further demonstrate the sensitivity and efficacy of your FBP-based reporter system, I recommend using qPCR to analyze the expression levels of key jasmonate-responsive and salicylic acid-responsive genes. This approach would provide additional validation of the system's responsiveness to these signaling pathways.
4. In Figure S2, the corresponding labels for panels (a) and (b) appear to be missing from the figure legend. Could you please clarify or provide the necessary details for these panels?
5. What is the specific solvent in panel b of Figure S3? There is no relevant explanation in the annotation, and what is the purpose of this setting? Additionally, regarding the description of the WRKY70 part in this figure in the main text: "In the case of pWRKY70, luminescence of untreated samples was high, likely reflecting upregulation of WRKY70 following agrobacterial infection in our assay," how was this conclusion drawn? (I didn't quite understand this explanation...)
6. Please merge the annotations of Figure S7 that are split across two pages into one.

Reviewer #2

(Remarks to the Author)
Jasmonic acid and salicylic acids are two major hormones involved in plant response to pests and pathogens. In this manuscript, Balakireva et al. developed autoluminescent plants carrying pORCA3 or pWRKY70-driven autoluminescent reporters which respond to JA and SA respectively. Imaging of Arabidopsis and Nicotiana benthamiana reporter lines in response to attacks of pests and pathogens showed that the responses of the reporters are consistent with the dynamics of SA and JA reported in the literature.

1. One key issue with transcription-based reporters is the specificity of the promoters used. No evidence was shown that pORCA3 and pWRKY70 are only responsive to SA and JA.
2. The spatial and temporal changes in JA and SA concentrations have been studied extensively using other approaches (reporters and quantification of the hormone levels). The manuscript has not sufficiently demonstrated the usefulness of pORCA3 or pWRKY70 reporter lines in obtaining new data.

Reviewer #3

(Remarks to the Author)
Following their breakthrough report on developing autoluminescent plants by overexpressing Fungal Bioluminescent Pathway (FBP) genes in Nicotiana (Mitiouchkina 2020 Nat Biotechnol), herein the authors have adapted the system to engineer Arabidopsis thaliana and Nicotiana benthamiana transcriptional reporters that respond to pathogen attacks and mechanical wounding. Induced reporter autouminescence could be easily detected with non-invasive consumer-grade cameras (eg. smartphone), rendering the reporter system readily available across laboratory and natural conditions, as well as amenable to further applications. The topic is timely, exciting and of broad general interest.

The strength and novelty of this work lies predominantly in identifying which of the 5 FBP genes (HispS, NpgA, H3H, Luz, or CPH) can be placed under the control of an inducible promoter instead of a constitutive one (p35S). The authors settled on exchanging the promoter for Luz with either Jasmonate (JA) or Salycilic Acid (SA) inducible ones, with favourable dynamic ranges and signal-to-noise ratios.

At the same time, the specificity of jasmonate (JA) and salicylic acid (SA) promoters have not been tested in sufficient detail to substantiate the claims of the autoluminescent reporters serving as hormone reporters. Here are a few suggestions to improve this work:

MAJOR ISSUES
- It remains unclear if pORCA3 from Catharanthus roseus is JA-specific in A. thaliana and N. benthamiana. In fact, Memelink et al 2002 reported pORCA3 is controlled by ERF Transcription factors as well, and is hence potentially responding to Ethylene. To validate the JA-specificity of this promoter, the authors should transform their constructs in JA deficient (eg. aos) or signalling mutants (eg. coi1). If pORCA3 results to be not JA-specific, there are many others well studied in Arabidopsis.
- The same should be also done to validate the specificity of pWRKY70 towards SA (eg. eds5, ics1, pal1 mutants). Overall, the activation dynamics of these two promoters seem quite late and may be regulated by other processes. For example, the eFP browser shows that WRKY70 can be induced by UVB, CHX, BL, salt, etc (and is also induced during Agrobacterium transformation herein). It is therefore important for the authors to verify if these promoters are really the best option to report on specific JA or SA processes.
- The authors should include more information in the main text on the temporal activation dynamics in response to different stresses in the two plant species. For example, how fast can the reporters respond to wounding or pathogen attack, and for how long are they active after the stimulus has ceased?
- The manuscript would benefit by extending the data shown and increasing the number of figures. Many of the supplementary data should in fact be presented as main figures. Separating the figures between JA and SA reporters, in the 2 plant species after wounding and pathogen attack would also increase clarity. Mock control inoculations should be included in every experiment
- In their previous work (Mitiouchkina 2020 Nat Biotechnol) the authors showed that the overexpression of 4 FBP genes (HispS, H3H, Luz, or CPH) did not alter plant growth nor other measurable phenotypes. In this work, a 5th gene (NpgA) has been included, similarly to (Khakhar et al 2020 elife). The authors should report if this impacts any phenotype in the two plant species

OTHER POINTS
- ‘In plant cell packs made of Nicotiana tabacum BY-2 cells 30 , luminescence increased in most pathway designs upon induction’ Sentence unclear, rephrase
- methods for hormone quantification are not described in sufficient detail (eg. which internal standard was used? Please provide references), and should be reported as mol/g FW (not peak area)

Version 1:

Reviewer comments:

Reviewer #1

(Remarks to the Author)
This revised manuscript presents a significant improvement over the previous version. The authors have thoroughly addressed my concerns by supplementing key experimental data and refining the text, resulting in a more coherent and convincing narrative. The development of a dual-responsive biosensor system using the FBP platform to non-invasively visualize salicylic acid and jasmonic acid signaling represents a notable technical advance. The use of fungal genes and stress-responsive promoters demonstrates innovative design, and the validation in planta during biotic stress conditions confirms the sensor’s sensitivity and functionality. The work provides valuable insights into plant defense mechanisms and

offers a robust conceptual foundation for future smart sensor applications. I am satisfied with the revisions and support the publication of the manuscript in its current form.

Reviewer #2

(Remarks to the Author)
My concerns have been adequately addressed.

Reviewer #3

(Remarks to the Author)
Overall, the development of bioluminescence-based SA and JA reporters is exciting and meaningful, but requires a more thorough validation of promoter specificity and a more detailed spatiotemporal description of the reporters' behaviour. In this revised manuscript, the authors did not address my major previous concerns in sufficient detail. Contrarywise, some of the new data only strengthens previous issues. Specifically:

1. The authors keep WRKY70 and ORCA3 promoters in the main figures, even if the new data shows they are not specific for SA and JA (SFig.7/19/20). To comply with the reviewers' requests, they include additional promoters in BY-2 cells but not in plants (SFig22). The hormone-dependency and specificity of both SA and JA promoters should be thoroughly validated by evaluating reporter behaviour in relevant hormone-deficient mutant backgrounds, especially in Arabidopsis (Rev3, major issue 1 & Rev2, point2)

2. Only after validating the specificity of SA and JA promoters can the authors perform a valid spatiotemporal analysis (Rev3 & Rev2, point2)

3. The title of this work is 'non-invasive imaging of SA and JA activities in planta' and in the introduction the authors ask 'How active are these hormones in different plant tissues in various ecological contexts, how local is their action, and how does it develop over time?'. These are not 'proof-of concept' questions on using bioluminescence reporters, but represent still unsolved biological issues. As stated previously, the manuscript would benefit from a major rearrangement by extending main figures and performing an accurate comparison on what are the spatiotemporal dynamics of reporter activation. Many critical data are embedded in the Supplement, precluding clarity and readability of the manuscript. On the other hand, if the authors insist on their proof of concept strategy of identifying the optimal architecture for bioluminescence engineering, the title and main text should be significantly revisited, with the consequence of lowering the potential impact of the study.

OTHER POINTS
- SFig.7 statistic analysis is missing, and PR4 is not a prominent MeJA responsive gene. Please test a transcript with higher expected MeJA-dependent inducibility (eg. JAZ transcripts)

Version 2:

Reviewer comments:

Reviewer #3

(Remarks to the Author)
In this third round of review, my main concern of using inadequate promoters for reporting on SA and JA pathways is still not addressed. At the same time, this work is novel and exciting, and the authors have done a fantastic work in optimizing the fungal bioluminescence pathway for plant expression

I support the authors willingness to rephrase the title, and tone down main conclusions on promoters used for SA and JA while highlighting limitations and future applications

I have no further comments

Open Access This Peer Review File is licensed under a Creative Commons Attribution 4.0 International License, which permits use, sharing, adaptation, distribution and reproduction in any medium or format, as long as you give appropriate credit to the original author(s) and the source, provide a link to the Creative Commons license, and indicate if changes were made.
In cases where reviewers are anonymous, credit should be given to 'Anonymous Referee' and the source.
The images or other third party material in this Peer Review File are included in the article's Creative Commons license, unless indicated otherwise in a credit line to the material. If material is not included in the article's Creative Commons license and your intended use is not permitted by statutory regulation or exceeds the permitted use, you will need to obtain permission directly from the copyright holder.
To view a copy of this license, visit https://creativecommons.org/licenses/by/4.0/

REVIEWER COMMENTS

Reviewer #1 (Remarks to the Author):

In this intriguing manuscript titled "Non-invasive Imaging of Salicylic and Jasmonic Acid Activities in Planta," the authors developed a reporter system based on the FBP (fluorescent biosensor protein) platform. They reconstituted the overexpression of four fungal genes—HispS, NpgA, H3H, and CPH—and utilized the jasmonate-responsive pORCA3 promoter and the salicylic acid-responsive pWRKY70 promoter to drive the expression of Luz. This enabled the transgenic plants to monitor defense responses during pest and pathogen attacks. The proposed plant sensor demonstrates high sensitivity and efficacy, offering a novel conceptual framework for smart plant sensors. Overall, the manuscript presents compelling findings. However, certain issues need to be addressed to further substantiate the conclusions and enhance the robustness of the study.

1. Salicylic acid (SA) and jasmonic acid (JA) are pivotal plant hormones that regulate defense responses and developmental processes. Since the FBP module is integrated into the phenylpropanoid metabolic pathway, and both SA and JA are known to modulate this pathway, it is crucial to address how FBP-based reporters differentiate between phenylpropanoid metabolism (e.g., caffeic acid production) and biotic stress signaling (e.g., WRKY70 promoter-driven Luz expression) in response to pest and pathogen attacks. Elucidating the effects of SA and JA on the phenylpropanoid pathway could provide valuable insights into the utility of FBP-based reporters for monitoring plant defense under biotic stress conditions.

We fully agree with the reviewer's comment that light emission depends not only on gene expression, but also on metabolic activity of the tissue. In fact, we acknowledge this as a fundamental limitation of our reporters in the last paragraph of the manuscript:

> *Although approaches based on autoluminescence have drawbacks, such as dependence of signal on metabolic activity of a tissue, they can provide insightful observations that may be further tested by orthogonal approaches.*

In our manuscript, we addressed monitoring metabolic changes by including in every experiment plants expressing the fungal bioluminescence pathway constitutively. These plants served as a control to monitor changes in metabolism happening under the very same experimental conditions.

To illustrate this, **Figures 1d** and **1e** now show that the luminescence intensity of *p35S-nnLuz* plants upon treatment with methyl jasmonate (JA) and salicylic acid (SA), respectively, is not significantly different from untreated controls. Similarly, **Supplementary Figure 12** shows the response of these plants to a range of stresses: mechanical wounding and infection with *Pectobacterium carotovorum*, *Pseudomonas savastanoi*, *Agrobacterium tumefaciens*, and herbivory by whiteflies. No notable luminescent response was observed in *p35S-nnLuz* plants under any of these conditions, in contrast to the pronounced responses observed in SA- and JA-responsive reporter lines (**Supplementary Figures 13** and **14**, respectively). This suggests that the constitutively expressed fungal bioluminescent system did not respond to SA- or JA-specific stimuli, supporting the conclusion that phenylpropanoid metabolism remains largely unaffected under these treatments, or at least not in a way that alters the output of the constitutive bioluminescence system.

To further test whether observed light emission in SA- and JA-responsive reporter lines is dependent on the synthesis of new luciferase protein molecules, for this revision, we conducted additional experiments. We treated plants with cycloheximide (CHX), a translation inhibitor. If luminescence were driven by changes in the phenylpropanoid metabolism alone, we would expect light emission to persist in the presence of CHX. If,

however, luminescence depends on newly synthesised proteins, CHX treatment would abolish the response. As shown now on Supplementary Figure 17, the transcript levels of both the luciferase and pathway marker gene (*PR-1a* as a marker for SA pathway, and *PR4* as a marker gene for JA pathway) increase upon SA or methyl jasmonate (MeJA) treatment, even in the presence of CHX, indicating that transcriptional activation does occur. However, luminescence only increases when the inducer is applied without CHX, demonstrating that reporter activity is dependent on *de novo* protein synthesis. These results support the conclusion that the reporter luminescence reflects hormone-induced gene expression, rather than fluctuations in phenylpropanoid metabolism.

CHX on its own—without SA or MeJA—enhanced the expression of both *pWRKY70-nnLuz* and *pORCA3-nnLuz*. This corroborates also Reviewer 3's note that the *WRKY70* promoter is CHX-inducible, and our results show that the *ORCA3* promoter is likewise responsive to CHX. Notably, adding MeJA in the presence of CHX produced a greater increase in luciferase activity.

Overall, we believe that while the reporters presented in our study are inherently dependent on metabolic activity, inclusion of constitutively luminescent control lines allows to control for metabolic effects. Our further qPCR and cycloheximide tests show that observed responses reflect hormonal activation, not metabolic changes.

2. In Figure 2A and 2B, the current line colors in the line chart are challenging to distinguish. To improve clarity and visual accessibility, it is recommended to modify the color scheme to ensure better differentiation between the lines.

Thank you for highlighting this – we've updated the colours in the revised manuscript.

3. Based on the pest and pathogen signaling pathways, you selected the jasmonate-responsive promoter pORCA3 and the salicylic acid-responsive WRKY70 promoter to drive the expression of Luz. To further demonstrate the sensitivity and efficacy of your FBP-based reporter system, I recommend using qPCR to analyze the expression levels of key jasmonate-responsive and salicylic acid-responsive genes. This approach would provide additional validation of the system's responsiveness to these signaling pathways.

We agree with the reviewer and thank for the suggestion. In the qPCR experiment performed for this revision (Supplementary Figure 7), we used *PR-1a* gene from *Nicotiana benthamiana* as a marker gene for salicylic acid pathway [1], and *PR4* from *Nicotiana benthamiana* [2] as a marker gene of jasmonic acid signaling. As a housekeeping gene, we used *EF-1a* from *Nicotiana benthamiana* [1].The experiment showed that corresponding inducers activated expression of both the hormone-sensitive promoters and the pathway marker genes *PR-1a / PR4.* Specifically, induction of *pWKRY70*-reporting plants with salicylic acid led to a 23-fold activation of expression of *PR-1a*, and a 63-fold activation of expression of *pWRKY70*-driven *Luz*. In *pORCA3*-reporting plants, a similar induction with methyl jasmonate led to a 3-fold activation of expression of *PR4*, and a 2-fold activation of expression of *pORCA3*-driven *Luz*.

In addition, to obtain information on the possible changes in the JA- and SA-stimulated gene expression in our marker lines, we also analyzed by qPCR the transcription of a set of key genes involved in the JA and SA pathways in *Arabidopsis thaliana* Col-0, *ORCA3*, and *WRKY70*. The results **(Supplementary Figure 5)** indicate that JA- and SA-specific pathways are not activated in the reporter lines, compared to the Col-0 line.
We believe overall, the data show that in our experimental conditions, we do observe activation of salicylic acid and jasmonic acid-specific pathways under relevant treatments.

4. In Figure S2, the corresponding labels for panels (a) and (b) appear to be missing from the figure legend. Could you please clarify or provide the necessary details for these panels?

Thank you, we corrected the figure legend.

5. What is the specific solvent in panel b of Figure S3? There is no relevant explanation in the annotation, and what is the purpose of this setting?

Thank you for noticing this, it was water, we included samples treated with water as a mock control. This has now been reflected in the description of the Supplementary Figure 3.

Additionally, regarding the description of the WRKY70 part in this figure in the main text: "In the case of pWRKY70, luminescence of untreated samples was high, likely reflecting upregulation of WRKY70 following agrobacterial infection in our assay," how was this conclusion drawn? (I didn't quite understand this explanation...)

In our experiment, plant cells transiently expressing *pWRKY70-nnLuz* showed high levels of luminescence even without the inducer (exogenous salicylic acid). Our interpretation is that plant cells respond to agrobacterial infiltration itself and activate the pathway endogenously, in line with what has been shown in the literature [3]. This also agrees with our results on stable lines obtained later in this work (Supplementary Figure 13a, panel "*Agrobacterium tumefaciens*"). We have now corrected the sentence: "In the case of *pWRKY70*, we observed high luminescence in untreated samples. It likely reflects the upregulation of *pWRKY70* in response to *Agrobacterium* infiltration during our assay [3]."

6. Please merge the annotations of Figure S7 that are split across two pages into one.

Thank you, we corrected the annotations of Figure S7.

Reviewer #2 (Remarks to the Author):

Jasmonic acid and salicylic acids are two major hormones involved in plant response to pests and pathogens. In this manuscript, Balakireva et al. developed autoluminescent plants carrying pORCA3 or pWRKY70-driven autoluminescent reporters which respond to JA and SA respectively. Imaging of Arabidopsis and Nicotiana benthamiana reporter lines in response to attacks of pests and pathogens showed that the responses of the reporters are consistent with the dynamics of SA and JA reported in the literature.

1. One key issue with transcription-based reporters is the specificity of the promoters used. No evidence was shown that pORCA3 and pWRKY70 are only responsive to SA and JA.

Thank you for this comment. We acknowledge that *pORCA3* and *pWRKY70* are not only responsive to SA and JA, and we should have made this clearer in the manuscript. Besides salicylic acid, the *WRKY70* promoter is known to respond to PAMP flg22 [4], brassinolide [5], systemic resistance signal N-hydroxy-pipecolic acid [6], and abiotic cues such as drought [7,8] and salt stress [7]. Jasmonates activate the *ORCA3* promoter, but it is also known to be affected by UV-B irradiation [9], fungal-type elicitors such as β-cyclodextrin [10,11], crude pathogen extracts that trigger Ca^{2+}-dependent phosphorylation cascades [12], and prolonged dark exposure/light shifts [10]. We now included this information in the manuscript, and provided a separate Supplementary Table 3 summarising available literature on the responses of these promoters to various treatments.

To address this point in more detail, for this revision we assessed the specificity of the *WRKY70* and *ORCA3* promoters by treating *Nicotiana benthamiana* leaves with high concentrations of phytohormones and their analogues or precursors (**Supplementary Figure 19**): abscisic acid (ABA, 100 µM), coronatine (10 µM), 1-naphthaleneacetic acid (NAA, 100 µM), thidiazuron (100 µM), 24-epibrassinolide (10 µM), gibberellic acid GA_3 (10 µM), the synthetic strigolactone GR24 (10 µM) and 1-aminocyclopropane-1-carboxylic acid (ACC, 100 µM); salicylic acid (SA, 100 µM) and jasmonoyl-isoleucine (JA-Ile, 1 mM) served as positive controls. *pWRKY70-nnLuz* plants only responded to salicylic acid, while pORCA3-nnLuz plants in addition to activation upon treatment with isoleucine jasmonate, were also activated by the epibrassinolide and GA_3.

Parallel experiments in *Arabidopsis thaliana* reporter lines (**Supplementary Figure 20**) used 1-naphthaleneacetic acid (NAA, 500 nM), 1-aminocyclopropane-1-carboxylic acid (ACC, 1 µM), 6-benzylaminopurine (BAP, 500 nM), aminoethoxyvinylglycine (AVG, 1 µM), indole-3-acetic acid (IAA, 500 nM), methyl jasmonate (MeJA, 500 nM and 5 µM), SA (500 nM and 5 µM) and trans-zeatin (500 nM). In addition to the expected activation by methyl jasmonate, IAA and NAA caused a luminescence increase in *pORCA3-nnLuz* plants. We believe that direct activation of *ORCA3* promoter by auxins has not been previously demonstrated in *A. thaliana*, although hormonal cross-talk and indirect regulatory connections have been noted. One example is the identification of a SHI/STY-family transcription factor in *C. roseus* that binds to the *ORCA3* promoter. The SHI/STY family (exemplified by *Arabidopsis* STYLISH1, STY1) is known to influence auxin biosynthesis and organ development. In a yeast one-hybrid screen, authors found a *Catharanthus* SHI/STY factor binding the *ORCA3* promoter, suggesting a potential link between auxin-related regulators and *ORCA3* control[13].

Overall, both the literature and our results show limited specificity of the selected promoters, and we have now updated the main text to highlight reporters' limitations to the readers and encourage usage of appropriate controls. As we discuss at the end of the manuscript, autoluminescent reporters presented here can be used to obtain insights into plant physiology, assuming that such insights are *verified with an orthogonal approach* in follow-up experiments.

More specific promoters can be used to drive Luz expression and it is straightforward to construct new reporter lines based on the luciferase-less master lines NB237 and AT8462 reported in our paper. To illustrate this, for the revised version of this manuscript, we assembled another jasmonic acid reporter based on the jasmonate-specific synthetic promoter *pJAZ1(x5)*. In BY-2 cell packs (Supplementary Figure 22), reporters based on this promoter showed low pre-induction signal, which was robustly elevated (50-fold) upon treatment with jasmonate isoleucine. In the same experiment, a *pORCA3*-based reporter showed a two orders of magnitude higher background luminescence and only a 6-fold elevation of the signal upon induction. We included the *pJAZ1(x5)*-based reporter into the Supplementary Table 1 and will make it available through Addgene, pending the approval from the North Carolina State University as per terms of the MTA.

Overall, we see our work largely as a proof-of-concept study aimed at (1) identifying an optimal architecture of the autoluminescent pathway for constructing molecular physiology reporters in plants, (2) demonstrating their ability to non-invasively report molecular events on consumer-grade equipment (which is inexpensive enough to allow running multiple months-long parallel experiments), and (3) providing validated luciferase-less master lines for further biosensor engineering.

2. The spatial and temporal changes in JA and SA concentrations have been studied extensively using other approaches (reporters and quantification of the hormone levels). The manuscript has not sufficiently demonstrated the usefulness of pORCA3 or pWRKY70 reporter lines in obtaining new data.

Discovering novel biology was not the primary goal of this study, even though we think we've made some interesting observations, e.g. showing dynamics of local salicylic acid activity in response to whitefly feeding. In all our efforts shown in this manuscript, we intended to show and defend a novel method for observing JA/SA signalling output that is simple, non-invasive, and allowing organism-scale imaging of molecular physiology. Our reporters allow for inexpensive time- and space-resolved imaging — not only in the lab, but also in the field setting, in a real ecological context. Alternative reporting technologies are either invasive (for example, mass-spectrometry), show low signal-to-noise ratio and need illumination (fluorescent-based reporters, due to autofluorescence of plant tissues), or require supply of luciferin substrate (traditional luciferase-based reporters) which is expensive and incompatible with field- or longitudinal studies.

Reviewer #3 (Remarks to the Author):

Following their breakthrough report on developing autoluminescent plants by overexpressing Fungal Bioluminescent Pathway (FBP) genes in Nicotiana (Mitiouchkina 2020 Nat Biotechnol), herein the authors have adapted the system to engineer Arabidopsis thaliana and Nicotiana benthamiana transcriptional reporters that respond to pathogen attacks and mechanical wounding. Induced reporter autoluminescence could be easily detected with non-invasive consumer-grade cameras (eg. smartphone), rendering the reporter system readily available across laboratory and natural conditions, as well as amenable to further applications. The topic is timely, exciting and of broad general interest.

The strength and novelty of this work lies predominantly in identifying which of the 5 FBP genes (HispS, NpgA, H3H, Luz, or CPH) can be placed under the control of an inducible promoter instead of a constitutive one (p35S). The authors settled on exchanging the promoter for Luz with either Jasmonate (JA) or Salycilic Acid (SA) inducible ones, with favourable dynamic ranges and signal-to-noise ratios.

At the same time, the specificity of jasmonate (JA) and salicylic acid (SA) promoters have not been tested in sufficient detail to substantiate the claims of the autoluminescent reporters serving as hormone reporters. Here are a few suggestions to improve this work:

MAJOR ISSUES
- It remains unclear if pORCA3 from Catharanthus roseus is JA-specific in A. thaliana and N. benthamiana. In fact, Memelink et al 2002 reported pORCA3 is controlled by ERF Transcription factors as well, and is hence potentially responding to Ethylene. To validate the JA-specificity of this promoter, the authors should transform their constructs in JA deficient (eg. aos) or signalling mutants (eg. coi1). If pORCA3 results to be not JA-specific, there are many others well studied in Arabidopsis.
- The same should be also done to validate the specificity of pWRKY70 towards SA (eg. eds5, ics1, pal1 mutants).

Thank you very much for these comments, which were also raised by Reviewer 2 and they are now discussed in our response to the Reviewer 2. We agree with the concern and acknowledge that these promoters are known to have non-specific activation by some inducers. Our new results on the **Supplementary Figures 8 and 9** also support this. Interestingly, we did not find *ORCA3* promoter to be inducible by ethylene precursor 1-aminocyclopropane-1-carboxylic acid (**Supplementary Figures 8**).

We also assembled another JA reporter using a different promoter (synthetic *pJAZ1(x5)* promoter). Its initial assessment in BY-2 cells showed a 50-fold activation upon treatment with isoleucine jasmonate, confirming that it is a promising candidate for thorough specificity assessment and generation of stable lines (**Supplementary Figures 22, 23**).

We have updated the main text to discuss non-specific responses of the reporters, and be cautious in the case of direct interpretation of autoluminescence experiments without orthogonal validation of results.

Overall, the activation dynamics of these two promoters seem quite late and may be regulated by other processes. For example, the eFP browser shows that WRKY70 can be induced by UVB, CHX, BL, salt, etc (and is also induced during Agrobacterium transformation herein). It is therefore important for the authors to verify if these promoters are really the best option to report on specific JA or SA processes.

Thank you for this comment. We have tested the response of the reporters to the conditions suggested by the reviewer. Treatment with 200 mM NaCl did not induce luminescence in either reporter line; UV-B exposure led to an increase in luminescence in *pWRKY70-nnLuz*-expressing plants and a weaker, yet detectable, increase in the case of *pORCA3-nnLuz* plants (Supplementary Figure 10). Treatment with cycloheximide (CHX, 0.1 mg/mL), an inhibitor of protein synthesis, led to the activation of transcription from the *WRKY70* promoter, in accordance with eFP browser data.

For this revision, we designed and assembled an expanded set of SA- and JA-responsive reporter constructs for their testing using the tobacco cell packs transient expression system

Jasmonic acid reporter:

- *JAZ1(x5)*

Salicylic acid reporters:

- *PR1(x3)*
- *p(At)EDS1*
- *p(At)PAD4*
- *p(At)PR1*
- *p(At)SAG101*

Our analysis of the basal expression of these reporters in *Nicotiana tabacum* BY-2 cell packs is shown in the Supplementary Figure 23. These constructs represent a starting point for the development of more selective hormone-specific reporters. As discussed above, *pJAZ1(x5)-nnLuz* genetic construct showed strong response to the treatment with isoleucine jasmonate and represents a promising candidate for further studies. The response of SA-sensitive promoters to SA could not be tested in our assay due to the SA pathway activation by agrobacteria, requiring generation of stable lines which we could not do within the timeline of this revision.

- The authors should include more information in the main text on the temporal activation dynamics in response to different stresses in the two plant species. For example, how fast can the reporters respond to wounding or pathogen attack, and for how long are they active after the stimulus has ceased?

We agree; we now included specific data on the activation dynamics of the reporters throughout the manuscript.

- The manuscript would benefit by extending the data shown and increasing the number of figures. Many of the supplementary data should in fact be presented as main figures. Separating the figures between JA and SA reporters, in the 2 plant species after wounding and pathogen attack would also increase clarity. Mock control inoculations should be included in every experiment

We appreciate Reviewer's point on expanding the main text figures. We see the paper largely as a proof-of-concept demonstration of the utility of such reporters for the broad scientific audience: we thus prefer to keep a short paper format and higher-level illustrative figures in the main text, providing a more specialised reader with the detailed figures and raw data in the supplementary material. We also re-checked the inclusion of mock controls which already have been included in every experiment in the original paper.

- In their previous work (Mitiouchkina 2020 Nat Biotechnol) the authors showed that the overexpression of 4 FBP genes (HispS, H3H, Luz, or CPH) did not alter plant growth nor other measurable phenotypes. In this work, a 5th gene (NpgA) has been included, similarly to (Khakhar et al 2020 elife). The authors should report if this impacts any phenotype in the two plant species

Following Khakhar et al 2020, we reported the addition of the phosphopantetheinyl transferase NpgA in our previous work (Shakhova et al., Nature Methods 2024)[14]. No noticeable adverse phenotypic changes were observed between the wild type and bioluminescent lines in that work (Supplementary Figure 23 in Shakhova et al). Similarly, Hao Du's group reported that bioluminescent plants expressing *NpgA* were metabolically indistinguishable from the wild type plants (Figure 4c in [15]).

OTHER POINTS

- 'In plant cell packs made of Nicotiana tabacum BY-2 cells 30 , luminescence increased in most pathway designs upon induction' Sentence unclear, rephrase

Thank you for your comment, the sentence was replaced with the following: "In plant cell packs made of *Nicotiana tabacum* BY-2 cells, luminescence increased in most reporter constructs upon induction (Figure 1b, Supplementary Figure 1)."

- methods for hormone quantification are not described in sufficient detail (eg. which internal standard was used? Please provide references), and should be reported as mol/g FW (not peak area)

Thank you, we have updated the Methods section with additional details.

References

1. Abbas, H. M. K. *et al.* Heterologous WRKY and NAC transcription factors triggered resistance in Nicotiana benthamiana. *J. King Saud Univ. Sci.* **32**, 3005–3013 (2020).
2. Kiba, A. *et al.* Phosphatidylinositol-phospholipase C2 regulates pattern-triggered immunity in Nicotiana benthamiana. *J Exp Bot* **71**, 5027–5038 (2020).
3. Raman, V. *et al.* Overexpression of VIRE2-INTERACTING PROTEIN2 in Arabidopsis regulates genes involved in Agrobacterium-mediated plant transformation and abiotic stresses. *Sci. Rep.* **9**, 1–13 (2019).
4. Activation of Defense Response Pathways by OGs and Flg22 Elicitors in Arabidopsis Seedlings. *Molecular Plant* **1**, 423–445 (2008).
5. Chen, J. *et al.* Arabidopsis WRKY46, WRKY54, and WRKY70 Transcription Factors Are Involved in Brassinosteroid-Regulated Plant Growth and Drought Responses. *Plant Cell* **29**, 1425–1439 (2017).
6. Website. https://www.biorxiv.org/content/10.1101/2024.04.23.590810v1.full.pdf?utm_source=chatgpt.com.
7. Chen, X., Zhang, T., Wang, H., Zhao, W. & Guo, Z. Transcription factor WRKY complexes in plant signaling pathways. *Phytopathol. Res.* **7**, (2025).
8. Li, S. *et al.* Exploring the mechanisms of WRKY transcription factors and regulated pathways in response to abiotic stress. *Plant Stress* **12**, 100429 (2024).
9. Environmental stress induced biosynthesis of plant secondary metabolites- transcriptional regulation as a key. *Crop Design* **4**, 100100 (2025).
10. Changes in medicinal alkaloids production and expression of related regulatory and biosynthetic genes in response to silver nitrate combined with methyl jasmonate in Catharanthus roseus in vitro propagated shoots. *Plant Physiology and Biochemistry* **132**, 623–632 (2018).
11. Memelink, J. & Gantet, P. Transcription factors involved in terpenoid indole alkaloid biosynthesis in Catharanthus roseus. *Phytochemistry Reviews* **6**, 353–362 (2007).
12. ORCAnization of jasmonate-responsive gene expression in alkaloid metabolism. *Trends in Plant Science* **6**, 212–219 (2001).
13. Eklund, D. M. *et al.* The Arabidopsis thaliana STYLISH1 protein acts as a transcriptional activator regulating auxin biosynthesis. *Plant Cell* **22**, 349–363 (2010).
14. Shakhova, E. S. *et al.* An improved pathway for autonomous bioluminescence imaging in eukaryotes. *Nat Methods* **21**, 406–410 (2024).
15. Ge, J. *et al.* Integration of biological and information technologies to enhance plant autoluminescence. *Plant Cell* **36**, 4703–4715 (2024).

Point-by-point response

Overall, the development of bioluminescence-based SA and JA reporters is exciting and meaningful, but requires a more thorough validation of promoter specificity and a more detailed spatiotemporal description of the reporters' behaviour. In this revised manuscript, the authors did not address my major previous concerns in sufficient detail. Contrarywise, some of the new data only strengthens previous issues. Specifically:

1. The authors keep WRKY70 and ORCA3 promoters in the main figures, even if the new data shows they are not specific for SA and JA (SFig.7/19/20).
[....]
The hormone-dependency and specificity of both SA and JA promoters should be thoroughly validated by evaluating reporter behaviour in relevant hormone-deficient mutant backgrounds, especially in Arabidopsis (Rev3, major issue 1 & Rev2, point2)
[....]
2. Only after validating the specificity of SA and JA promoters can the authors perform a valid spatiotemporal analysis (Rev3 & Rev2, point2)

As supported by the recent experiments with orthogonal methodology (RT-qPCR, LC-MS — Supplementary Figures 5, 7, 11, 20), our reporter plants do increase light emission upon activation of JA- and SA-driven pathways. Being based on native hormone-sensitive promoters, these reporters do respond to other stimuli too (Supplementary Figures 3, 5, 7–11). We do not claim strict specificity, and in contrast, highlight the limitations of the promoters we used, advising the reader to confirm any insights from imaging datasets with orthogonal approaches.

Our perspective is that a technology may have limitations and still be very useful, as long as users are aware of its limitations. While the promoters we selected for stable lines are certainly responsive by some other stimuli, in our confirmation experiments in stable lines, bioluminescence activation coincided with the activation of the corresponding hormonal pathway assessed with orthogonal methods — highlighting that imaging data will be useful in obtaining initial insights into plant hormone physiology.

We see our work as v1 of this technology, which optimises the genetic architecture of the pathway, and validates the technical feasibility of such imaging at the organism scale. It does have limitations, and the next version of the technology will allow for a more specific and more quantitative imaging. We have already demonstrated in this paper that similar reporters can be constructed based on synthetic promoters which lack non-specific transcription factor binding motifs. In our parallel work, we have developed a colour-shifted luciferase for ratiometric imaging, which reduces metabolism-related signal variation. Consolidation of these and other advances will result in v2 of this technology in a few years.

To comply with the reviewers' requests, they include additional promoters in BY-2 cells but not in plants (SFig22).

Yes, the Reviewer is correct, the new synthetic reporters created for the revision were only tested in BY-2 cells but not in plants. This was our effort to demonstrate to the reader that one can further refine reporter specificity via promoter engineering, if the native promoters are deemed too pleiotropic.

3. The title of this work is ‘non-invasive imaging of SA and JA activities in planta’ and in the introduction the authors ask ‘How active are these hormones in different plant tissues in various ecological contexts, how local is their action, and how does it develop over time?’. These are not ‘proof-of concept’ questions on using bioluminescence reporters, but represent still unsolved biological issues.

As stated previously, the manuscript would benefit from a major rearrangement by extending main figures and performing an accurate comparison on what are the spatiotemporal dynamics of reporter activation. Many critical data are embedded in the Supplement, precluding clarity and readability of the manuscript. On the other hand, if the authors insist on their proof of concept strategy of identifying the optimal architecture for bioluminescence engineering, the title and main text should be significantly revisited, with the consequence of lowering the potential impact of the study.

We appreciate Reviewer’s point that the paper starts by listing unresolved questions related to plant hormone biology, but then represents a largely engineering study. We now corrected this bit to:

> *Here, we performed a proof-of-concept study, adapting the pathway to create transcription-based autoluminescence reporters that visualise hormone activities in planta.*

We also appreciate the reviewer's point on the title of the work; we are happy to amend the title, for example, to “Organism-scale autoluminescence imaging of molecular physiology *in planta*“ or another appropriate title.

To recap our perspective, we believe that before this work, it was not obvious (1) what the optimal architecture of the bioluminescence pathway is, (2) whether endogenous reporter promoters will generate enough autoluminescence light in stable lines, (3) whether reliable imaging would be possible on consumer cameras, (4) whether the dynamic range would be large enough to meaningfully track plant responses at the organism level. All these questions have now been answered, and additionally plant lines and plasmid that we characterised have been made available to engineer reporters for potentially any transcription-based readout of interest. This is why this work is of broad interest to plant biologists, and where most value of this work lies.

In addition, our study contributes unique datasets, for example, month-long dynamics of pWRKY70 and pORCA3 promoter activities upon healthy *N.benthamiana* development (with 30 min resolution!), time-lapse imaging of plant local response to whitefly attack, and others. We find that observations made with these autoluminescence tools are in agreement with the literature, while advising to interpret results with caution and validate with orthogonal methodology.

We are grateful for Reviewer’s time considering the manuscript, especially for raising concerns about promoter specificity. Experiments performed to address some of these concerns have improved the paper. We now include extensive information on limitations of the promoters, provide confirmation experiments with LC-MS, and provide alternative reporters based on synthetic promoters.

OTHER POINTS
- SFig.7 statistic analysis is missing, and PR4 is not a prominent MeJA responsive gene. Please test a transcript with higher expected MeJA-dependent inducibility (eg. JAZ transcripts)

Apologies if we misunderstand, we believe that statistical analyses are already provided in Supplementary Figure 7 for comparisons between mock and SA- or MeJA-treated samples. In this revision, we have additionally

included statistics for the cycloheximide (CHX) experiments, explicitly comparing +CHX versus –CHX within each hormone condition.

Regarding the marker choice, we selected PR4 based on its previous characterisation as a jasmonate-responsive transcripts in *Nicotiana benthamiana* [5–7]. We considered the suggestion to analyze JAZ transcripts; our literature survey identified the *N. benthamiana* JAZ3 gene with an experimentally validated MeJA responsiveness [8]. We have now performed RT-qPCR experiments specific to NbJAZ3 in *N. benthamiana* and showed the inducibility of this gene in response by MeJa treatment the level of which was similar to our pORCA3-based reporter level of inducibility (new Supplementary Figure 7a).

1. Lehmann, S. *et al.* Novel markers for high-throughput protoplast-based analyses of phytohormone signaling. *PLoS One* **15**, e0234154 (2020).
2. Montiel, G., Zarei, A., Körbes, A. P. & Memelink, J. The Jasmonate-Responsive Element from the ORCA3 Promoter from Catharanthus roseus is Active in Arabidopsis and is Controlled by the Transcription Factor AtMYC2. *Plant Cell Physiol.* **52**, 578–587 (2011).
3. Gutierrez, L. *et al.* Auxin controls Arabidopsis adventitious root initiation by regulating jasmonic acid homeostasis. *Plant Cell* **24**, 2515–2527 (2012).
4. Grunewald, W. *et al.* Expression of the Arabidopsis jasmonate signalling repressor JAZ1/TIFY10A is stimulated by auxin. *EMBO Rep* **10**, 923–928 (2009).
5. Kiba, A. *et al.* Phosphatidylinositol-phospholipase C2 regulates pattern-triggered immunity in Nicotiana benthamiana. *J Exp Bot* **71**, 5027–5038 (2020).
6. Kiba, A. *et al.* SEC14 phospholipid transfer protein is involved in lipid signaling-mediated plant immune responses in Nicotiana benthamiana. *PLoS One* **9**, e98150 (2014).
7. Ueta, Y., Mizutani, Y., Ohnishi, K., Hikichi, Y. & Kiba, A. Phosphatidylinositol-phospholipase C1 negatively regulates the hypersensitive response in Nicotiana benthamiana. *Physiol. Mol. Plant Pathol.* **116**, 101724 (2021).
8. Yan, X. *et al.* NbJAZ3 is required for jasmonate-meditated glandular trichome development in Nicotiana benthamiana. *Physiol Plant* **174**, e13666 (2022).
